# Accelerated waning of the humoral response to COVID-19 vaccines in obesity

**DOI:** 10.1038/s41591-023-02343-2

**Published:** 2023-05-11

**Authors:** Agatha A. van der Klaauw, Emily C. Horner, Pehuén Pereyra-Gerber, Utkarsh Agrawal, William S. Foster, Sarah Spencer, Bensi Vergese, Miriam Smith, Elana Henning, Isobel D. Ramsay, Jack A. Smith, Stephane M. Guillaume, Hayley J. Sharpe, Iain M. Hay, Sam Thompson, Silvia Innocentin, Lucy H. Booth, Chris Robertson, Colin McCowan, Steven Kerr, Thomas E. Mulroney, Martin J. O’Reilly, Thevinya P. Gurugama, Lihinya P. Gurugama, Maria A. Rust, Alex Ferreira, Soraya Ebrahimi, Lourdes Ceron-Gutierrez, Jacopo Scotucci, Barbara Kronsteiner, Susanna J. Dunachie, Paul Klenerman, PITCH Consortium, Adrian J. Park, Francesco Rubino, Abigail A. Lamikanra, Hannah Stark, Nathalie Kingston, Lise Estcourt, Heli Harvala, David J. Roberts, Rainer Doffinger, Michelle A. Linterman, Nicholas J. Matheson, Aziz Sheikh, I. Sadaf Farooqi, James E. D. Thaventhiran

**Affiliations:** 1 University of Cambridge Metabolic Research Laboratories and NIHR Cambridge Biomedical Research Centre, Wellcome-Medical Research Council (MRC) Institute of Metabolic Science, University of Cambridge, Cambridge, UK; 2 MRC Toxicology Unit, University of Cambridge, Cambridge, UK; 3 Cambridge Institute of Therapeutic Immunology and Infectious Disease, University of Cambridge, Cambridge, UK; 4 Department of Medicine, University of Cambridge, Cambridge, UK; 5 Nuffield Department of Primary Care Health Sciences, University of Oxford, Oxford, UK; 6 Babraham Institute, Babraham Research Campus, Cambridge, UK; 7 NIHR Cambridge Clinical Research Facility, Cambridge University Hospitals NHS Foundation Trust, Cambridge, UK; 8 Department of Infectious Diseases, Cambridge University Hospitals NHS Foundation Trust, Cambridge, UK; 9 Cambridge Institute for Medical Research, University of Cambridge, Cambridge, UK; 10 Department of Mathematics and Statistics, University of Strathclyde, Glasgow, UK; 11 Usher Institute, University of Edinburgh, Edinburgh, UK; 12 Immunology, Cambridge University Hospitals NHS Foundation Trust, Cambridge, UK; 13 Clinical Biochemistry, Cambridge University Hospitals NHS Foundation Trust, Cambridge, UK; 14 Peter Medawar Building for Pathogen Research, Nuffield Department of Clinical Medicine, University of Oxford, Oxford, UK; 15 NDM Centre for Global Health Research, Nuffield Department of Medicine, University of Oxford, Oxford, UK; 16 NIHR Oxford Biomedical Research Centre, Oxford University Hospitals NHS Foundation Trust, Oxford, UK; 17 Translational Gastroenterology Unit, University of Oxford, Oxford, UK; 18 Mahidol-Oxford Tropical Medicine Research Unit, Bangkok, Thailand; 19 Department of Diabetes, King’s College London and Kingʼs College Hospital NHS Foundation Trust, London, UK; 20 NHS Blood and Transplant, Oxford, UK; 21 Radcliffe Department of Medicine, University of Oxford, Oxford, UK; 22 NIHR BioResource, Cambridge University Hospitals NHS Foundation Trust, Cambridge, UK; 23 NHS Blood and Transplant, London, UK; 24 NHS Blood and Transplant, Cambridge, UK

**Keywords:** Endocrine system and metabolic diseases, Viral infection

## Abstract

Obesity is associated with an increased risk of severe Coronavirus Disease 2019 (COVID-19) infection and mortality. COVID-19 vaccines reduce the risk of serious COVID-19 outcomes; however, their effectiveness in people with obesity is incompletely understood. We studied the relationship among body mass index (BMI), hospitalization and mortality due to COVID-19 among 3.6 million people in Scotland using the Early Pandemic Evaluation and Enhanced Surveillance of COVID-19 (EAVE II) surveillance platform. We found that vaccinated individuals with severe obesity (BMI > 40 kg/m^2^) were 76% more likely to experience hospitalization or death from COVID-19 (adjusted rate ratio of 1.76 (95% confidence interval (CI), 1.60–1.94). We also conducted a prospective longitudinal study of a cohort of 28 individuals with severe obesity compared to 41 control individuals with normal BMI (BMI 18.5–24.9 kg/m^2^). We found that 55% of individuals with severe obesity had unquantifiable titers of neutralizing antibody against authentic severe acute respiratory syndrome coronavirus 2 (SARS-CoV-2) virus compared to 12% of individuals with normal BMI (*P* = 0.0003) 6 months after their second vaccine dose. Furthermore, we observed that, for individuals with severe obesity, at any given anti-spike and anti-receptor-binding domain (RBD) antibody level, neutralizing capacity was lower than that of individuals with a normal BMI. Neutralizing capacity was restored by a third dose of vaccine but again declined more rapidly in people with severe obesity. We demonstrate that waning of COVID-19 vaccine-induced humoral immunity is accelerated in individuals with severe obesity. As obesity is associated with increased hospitalization and mortality from breakthrough infections, our findings have implications for vaccine prioritization policies.

## Abstract (web summary)

Epidemiological analyses coupled with immunological phenotyping suggest that humoral immunity induced by COVID-19 vaccines wanes more rapidly in individuals with severe obesity compared to individuals with a BMI within the normal range.

## Main

Globally, obesity (defined as body mass index (BMI) > 30 kg/m^2^) is a major risk factor for severe Coronavirus Disease 2019 (COVID-19)^1^. Severe obesity (BMI > 40 kg/m^2^), which affects 3% of the population in the United Kingdom (UK) and 9% in the United States (US) (https://www.worldobesity.org/), is associated with a 90% higher risk of death from COVID-19 (ref. ^2^). Obesity is associated with type 2 diabetes mellitus, hypertension, chronic kidney disease and heart failure, comorbidities that may independently increase the risk of severe COVID-19 (refs. ^3–7^).

COVID-19 vaccines reduce the risk of symptomatic infection, hospitalization and mortality due to COVID-19 (refs. ^8,9^). They generate antibodies against the spike (S) protein of severe acute respiratory syndrome coronavirus 2 (SARS-CoV-2), comprising S1 and S2 subunits; S1 contains the receptor-binding domain (RBD), which mediates binding of the virus to angiotensin converting enzyme-2 (ACE-2) on host cells. The RBD is the main target for SARS-CoV-2 neutralizing antibodies, which inhibit viral replication in vitro and correlate with protection against infection in vivo^10–12^. As well as neutralizing antibodies, non-neutralizing antibodies and cellular immunity contribute to protection, particularly against severe COVID-19. As immunity acquired after two doses of vaccine wanes over 6–9 months, many countries have elected to administer booster doses to maintain immune protection, particularly in older people and the immunocompromised^13,14^.

People with obesity have impaired immune responses to conventional influenza, rabies and hepatitis vaccines^15–18^; however, the effects of obesity on their responses to mRNA and adenoviral-vectored vaccines is not known. Several studies have suggested that, after COVID-19 vaccination, antibody titers may be lower in individuals with obesity than in the general population^19–24^. One possible explanation is the impact of needle length on vaccine dosing in individuals with obesity^25^, risking subcutaneous administration of a vaccine that is intended to be intramuscular. To date, longitudinal studies to investigate the duration of protection after COVID-19 vaccination in individuals with obesity have not been performed. Here we focus on individuals with severe obesity (those at highest risk). We conducted a prospective, longitudinal study that allowed us to demonstrate that, although initial and peak responses were similar in individuals with severe obesity and individuals with normal weight, there was accelerated decline in antibody levels over time that correlated with increased frequency of hospitalization and mortality from breakthrough infections. The findings and policy implications are summarized in Table 1.

**Table 1:** Policy summary

Background	Obesity is associated with increased hospitalization and mortality due to severe COVID-19. Although COVID-19 vaccines are highly effective, details of the immune response and duration of vaccine efficacy in individuals with obesity are unknown.
Main findings and limitations	Using real-time data collected on over 3.6 million people in Scotland who had received two doses of primary COVID-19 vaccine, we show that the risk of severe COVID-19 is markedly increased (76%) in individuals with severe obesity (BMI > 40 kg/m^2^). Breakthrough infections resulted in increased hospitalization and mortality due to COVID-19 and occurred more rapidly in individuals with severe obesity than in individuals with normal weight (after 10 weeks versus after 20 weeks), suggesting more rapid waning of protection. In an accompanying clinical study, we show that peak neutralizing antibody titers are similar in individuals with normal weight and individuals with severe obesity, indicating that the initial vaccine response is similar between the two groups. However, longitudinal immunophenotyping of both groups demonstrated that neutralizing capacity declines more rapidly in individuals with severe obesity. Although we did not observe an associated T cell defect, the number of individuals studied in the clinical cohort was modest, limiting the power to detect small differences.
Policy implications	Taken together, our results indicate that increased BMI affects the rate of decline of vaccine-mediated immunity against SARS-CoV-2 in the population. Given the high prevalence of obesity worldwide, these findings have major implications for vaccination policy globally. COVID-19 vaccines may need to be administered more frequently in individuals with severe obesity to achieve the duration of protection from severe COVID-19 that is seen in individuals with normal BMI. Furthermore, our demonstration that the kinetics of the adaptive immune response to vaccination differs in individuals with severe obesity has implications for immunization against other infectious diseases where the longitudinal vaccine response remains incompletely characterized. There is a pressing need to ensure appropriate demographic representation in clinical research studies and trials, which must seek to include individuals with varying degrees of obesity. Our work highlights the critical importance of collecting data on BMI and metabolic risk factors so that lessons can be learned rapidly to guide changes in policy and improve health outcomes.

## Results

### Severe COVID-19 outcomes in vaccinated individuals

**Fig. 1:**
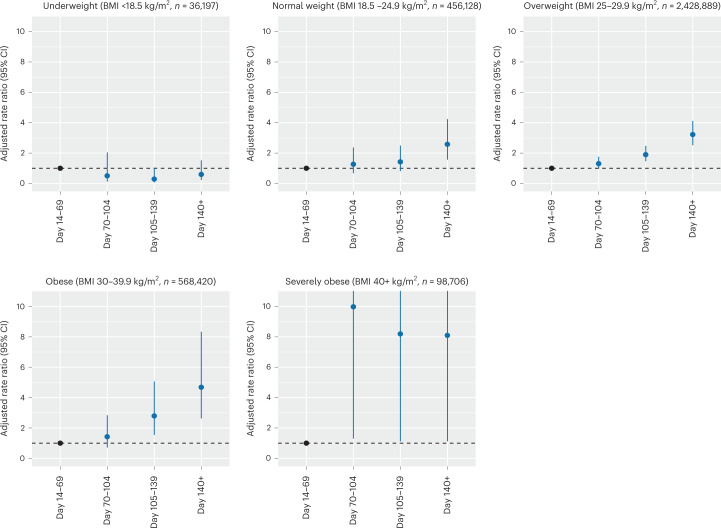
Risk of severe COVID-19 outcomes after primary vaccination and relationship with BMI. Panels depict the aRRs for hospitalization or death (severe COVID-19 outcomes) with time after the second COVID-19 vaccine dose for individuals in each BMI category in the EAVE-II cohort, Scotland. aRRs were estimated from five different models, one for each BMI category. aRRs were calculated against baseline risk at 14–69 d after the second vaccine dose. Error bars indicate 95% CIs. The number (*n*) of individuals in each BMI category is indicated. Hospitalization and mortality from breakthrough infections after the second vaccine dose presented more quickly in individuals with severe obesity (after 10 weeks; that is, 70 d) and obesity (15 weeks; that is; 105 d) than in individuals with normal weight (20 weeks; that is, 140 d). aRRs are provided as mean with 95% CIs. Source data

**Extended Data Fig. 1:**
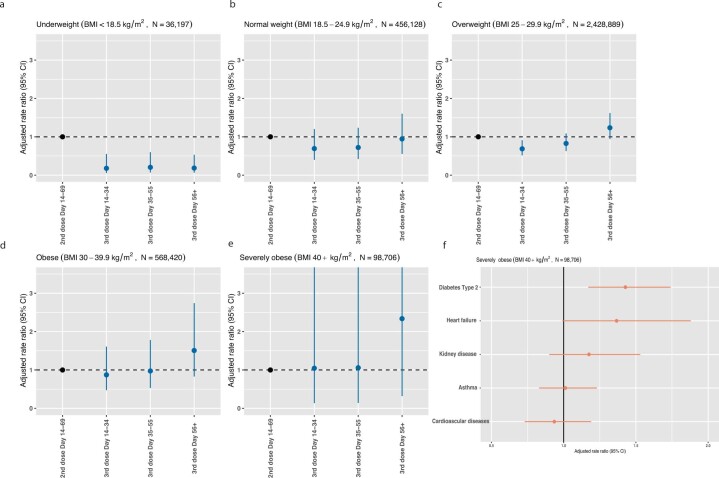
Adjusted rate ratios for hospitalization and death due to COVID-19 in vaccinated people. **a-e**, Adjusted rate ratios for hospitalization or death following third (booster) doses per BMI category (n = 36197 BMI < 18.5 kg/m^2^, n = 456128 BMI 18.5-24.9 kg/m^2^, n = 2428889 BMI 25-29.9 kg/m^2^, n = 568420 BMI 30-39.9 kg/m^2^, n = 98706 BMI > 40 kg/m^2^). Adjusted Rate Ratios (aRR) for hospitalisation or death following third (booster) dose for different body mass index (BMI) categories. Error bars indicate 95% confidence intervals. N indicates number of people in each category. **f**, adjusted rate ratios for hospitalisation and death in severely obese individuals with obesity-associated comorbidities (n = 98706 BMI > 40 kg/m^2^). aRR are provided as mean with error bars indicating 95% confidence intervals. Source data

**Extended Data Fig. 2:**
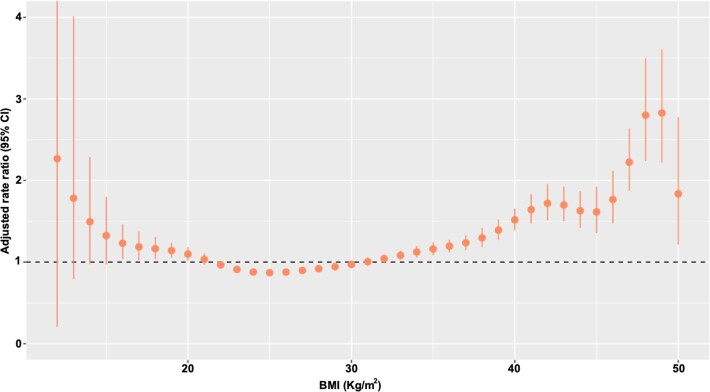
Adjusted rate ratios for hospitalization and death due to COVID-19 in vaccinated people using individual BMI values. Association between BMI (kg/m^2^) as a continuous trait and adjusted rate ratios (with 95% CI, confidence intervals) for severe COVID-19 outcomes. aRR are provided as mean with error bars indicate 95% confidence intervals.

To investigate the real-world effectiveness of COVID-19 vaccination in individuals with obesity, we used the Early Pandemic Evaluation and Enhanced Surveillance of COVID-19 (EAVE II) surveillance platform, which draws on near-real-time nationwide healthcare data for 5.4 million individuals (~99%) in Scotland, UK^26–29^. We interrogated data on 3,588,340 individuals aged ≥18 years who received a second dose (of the primary vaccination schedule) or a third booster dose of vaccine between 8 December 2020 and 19 March 2022 and were followed-up until hospitalization, death or the end of the study (19 March 2022; Table 2). BMI was recorded for 1,734,710 (49.2%) individuals who were included in the study. We first examined the effect of BMI on COVID-19-related hospitalization and mortality ≥14 d after receiving a second dose of either Pfizer-BioNTech BNT162b2 mRNA or AstraZeneca ChAdOx1 nCoV-19 vaccines. Between 14 September 2020 and 19 March 2022, there were 10,983 individuals (0.3%, 6.0 events per 1,000 person-years) who had a severe COVID-19 outcome: 9,733 individuals were hospitalized and 2,207 individuals died due to COVID-19 (957 individuals were hospitalized before their death). Individuals with severe obesity (BMI > 40 kg/m^2^) were at increased risk of severe COVID-19 outcomes after a second vaccine dose compared to those with BMI in the normal range, with an adjusted rate ratio (aRR) of 1.76 (95% confidence interval (CI), 1.60–1.94) after adjusting for age, sex and socioeconomic status (Methods, Supplementary Data Tables 1–7 and Extended Data Figs. 1 and 2). A modest increase in risk was also seen in individuals who were obese (BMI 30–40 kg/m^2^) and those who were underweight (BMI < 18.5 kg/m^2^) (aRR 1.11, 95% CI 1.05–1.18 and aRR 1.28, 95% CI 1.12–1.47, respectively) (Supplementary Table 1). Individuals with obesity and severe obesity were at higher risk of hospitalization or death from COVID-19 after both a second (Fig. 1) and a third (booster) dose (Extended Data Fig. 1). Hospitalization and mortality from breakthrough infections after the second vaccine dose presented sooner in individuals with severe obesity (after 10 weeks; that is, 70 d) and obesity (after 15 weeks; that is, 105 d) than in individuals with normal weight (after 20 weeks; that is, 140 d) (Fig. 1 and Extended Data Fig. 1). A modest increase in risk was also seen for males in comparison to females (males versus females aRR 1.31, 95% CI 1.26–1.34).

**Table 2:** Population characteristics of individuals from EAVE II who received at least the second (of the primary vaccination schedule) or a third dose of a COVID-19 vaccine

Characteristic		Total vaccination (*n*, %)	Severe COVID-19 outcome (*n*, rate per 1,000 person-years)
Total		3,588,340 (100.0)	10,938 (6.0)
Sex	Female	1,879,578 (52.4)	5,528 (5.7)
	Male	1,708,762 (47.6)	5,455 (6.2)
Age group (years)	18–49	1,634,424 (45.5)	2,217 (2.5)
	50–64	1,021,352 (28.5)	2,447 (4.6)
	65–79	706,617 (19.7)	3,331 (9.2)
	80+	225,947 (6.3)	2,988 (26.8)
BMI (kg/m^2^)	<18.5	36,197 (1.0)	252 (13.7)
	18.5–24.9	456,128 (12.7)	1,813 (7.7)
	25–29.9	2,428,889 (67.7)	5,599 (4.5)
	30–39.9	568,420 (15.8)	2,710 (9.3)
	40+	98,706 (2.8)	609 (12.2)

The frequency and rate per 1,000 person-years of severe COVID-19 outcomes (COVID-19-related hospitalization or death) was calculated. aRRs were estimated adjusting for all confounders, including age, sex, Scottish Index of Multiple Deprivation (SIMD), time since receiving the second dose of vaccine, number of pre-existing comorbidities, the gap between vaccine doses, previous history of SARS-CoV-2 infection and calendar time. Where the BMI was missing, it was imputed using ordinary least squares regression with all other independent variables included as predictors (BMI (imputed)).

Post hoc sensitivity analyses in clinically confirmed cases and after pooling the results of 10 imputations by chained equations generated very similar results (Supplementary Data Table 1). We found that vaccinated individuals with severe obesity and who had type 2 diabetes were at increased risk of admission to hospital or death due to COVID-19 (aRR 1.43, 95% CI 1.17–1.74; Extended Data Fig. 1f). A diagnosis of type 2 diabetes was independently associated with an increased risk of a severe COVID-19 outcome despite vaccination (aRR 1.13, 95% CI 1.07–1.19), although this was less than the risk associated with severe obesity. The aRR for type 2 diabetes was reduced slightly after adjusting for BMI (1.06, 95% CI 1.00–1.12).

### Immunity after primary vaccination in individuals with obesity

**Fig. 2:**
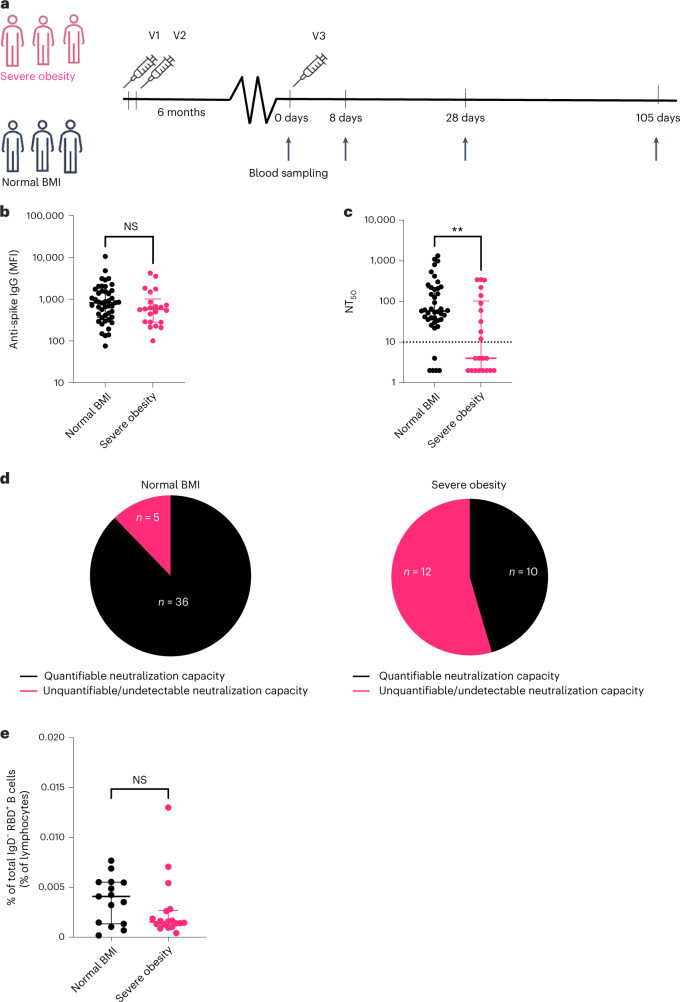
COVID-19 vaccine-induced immunity in individuals with severe obesity and individuals with normal weight 6 months after primary vaccination. **a**, Detailed longitudinal immunophenotyping studies were performed on individuals with severe obesity (magenta, *n* = 22) and normal BMI control individuals (black, *n* = 46). Samples were obtained 6 months after the second dose of COVID-19 vaccine (V2) administered as part of their primary vaccination course and at several timepoints after the third dose (V3) as indicated. **b**, Anti-spike IgG titers are similar in individuals with severe obesity (*n* = 22, magenta) and individuals with normal weight (*n* = 41, black) 6 months after primary vaccination course. Horizontal bars indicate the median and interquartile range. **c**, NT_50_ against wild-type SARS-CoV-2, with the dotted line indicating the limit of quantification. Horizontal bars indicate the median and interquartile range. **P = 0.061 in two-sided Mann–Whitney test. **d**, Proportion of individuals in both groups with unquantifiable or undetectable versus quantifiable titers of neutralizing antibodies. **e**, Frequency of antigen-experienced (IgD^−^) RBD-binding (RBD^+^) B cells 6 months after the primary vaccination course. Data are expressed as a percentage (%) of the total number of lymphocytes in individuals with severe obesity (*n* = 18, magenta) and individuals with normal weight (*n* = 15, black). Horizontal bars indicate the median and interquartile range. *P* values are from Mann–Whitney *U*-tests. NS, not significant. Source data

**Extended Data Fig. 3:**
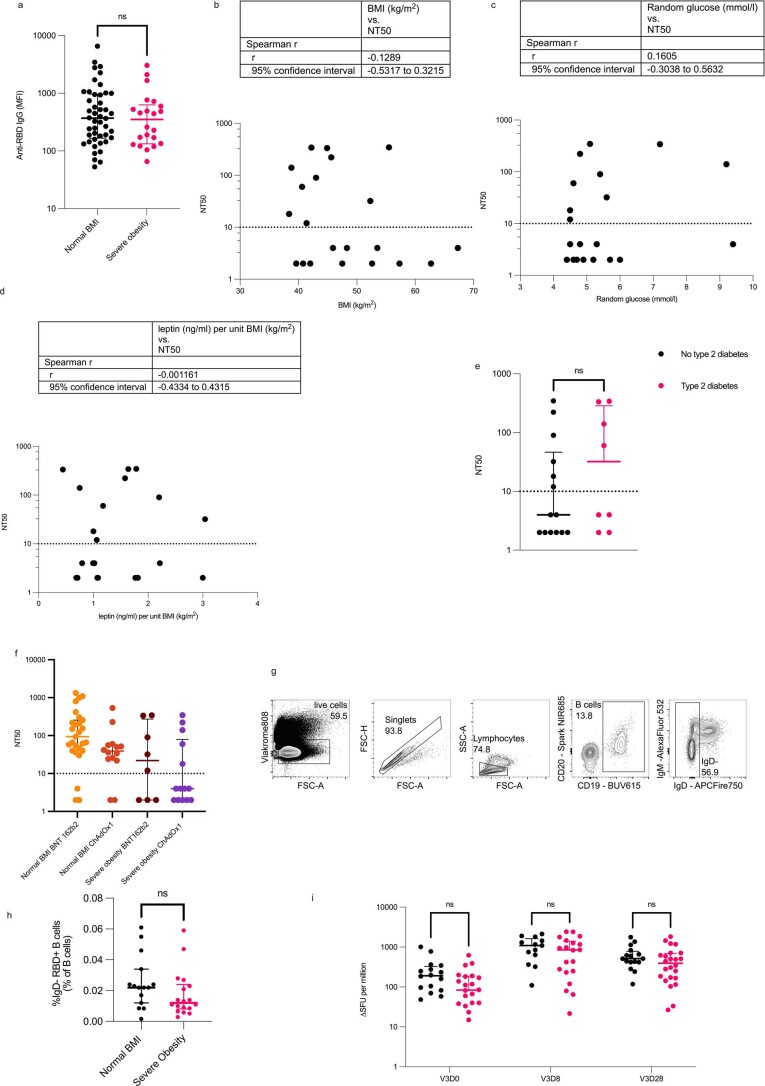
SARS-CoV-2 vaccine-induced immunity in people with severe obesity and normal BMI. **a**, Anti-RBD (receptor binding domain) IgG titres are comparable in 6 months after primary vaccination course (P = 0.5801 in Mann-Whitney U test) in people with severe obesity (N = 22) and normal BMI (46). **b-e**, Correlation between body mass index (BMI), random blood glucose, leptin levels per unit BMI in people with severe obesity (n = 22) and the presence (=8) or absence of type 2 diabetes (n = 14) and neutralizing capacity (NT50) 6 months after second dose. Non-parametric Spearman’s Rho correlations were calculated between NT50 and clinical parameters. Dotted line indicates the limit of quantification. A Mann-Whitney test was used to compare people with severe obesity with and without diabetes mellitus. **f**, Neutralizing capacity (NT50) 6 months after second dose by primary vaccination course - Pfizer-BioNTech BNT162b2 mRNA or AstraZeneca ChAdOx1. Dotted line indicates the limit of quantification. **g**, shows the B cell Pre-gating used in the flow cytometry analysis. **h**, shows the frequency of antigen-experienced (IgD-) Receptor Binding Domain binding (RBD + ), 6 months after the primary vaccination course; data expressed as a percentage (%) of the total number of B-cells in people with severe obesity (n = 15) and normal BMI (n = 18). Compared in Mann-Whitney U test. **i**, Antigen-specific T cell responses were measured by ELISpot. Interferon gamma spot forming units (SFU) were quantified. Analysed in mixed effects model. Each symbol represents an individual person and line indicates the median with interquartile range; black symbols indicate people with normal BMI (n = 16) and magenta people with severe obesity (n = 22). Ns, not significant. V3D0 is before third dose vaccination (V3), V3D8 is 8 days after third dose and V3D28 is 28 days after third dose vaccination. Source data

**Extended Data Fig. 4:**
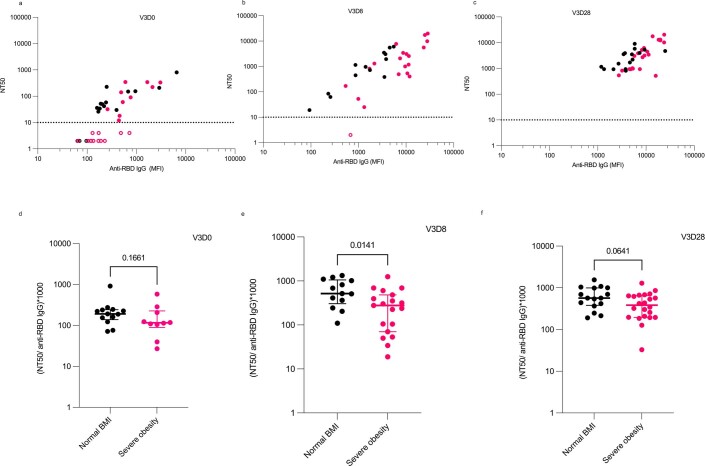
Relationship between anti-RBD IgG antibody levels and neutralizing capacity. **a-c**, Correlation between NT50 and anti-RBD IgG (MFI) before (V3D0, **a**), 8 days after (V3D8, **b**) and 28 days after (V3D28, **c**) third dose booster vaccination. **d-f**, Ratio of (NT50/anti-RBD IgG (MFI))*1000 before (V3D0, **d**), 8 days after (V3D8, **e**) and 28 days after (V3D28, **f**) third dose booster vaccination. Each symbol represents an individual person (controls with normal BMI (n = 16), black; severe obesity (n = 22), magenta). Open symbols represent individuals with unquantifiable or undetectable neutralizing activity. For **d-f**, median values and interquartile ranges are shown. P-values from Mann-Whitney U tests. Source data

To investigate the effects of severe obesity on humoral and cellular immunity to COVID-19 vaccination, we performed prospective longitudinal immunophenotyping of a clinical cohort of individuals with severe obesity (*n* = 28) and normal BMI control individuals (*n* = 41) in Cambridge, UK (SARS-CoV2 Vaccination Response in Obesity (SCORPIO) study) (Fig. 2 and Supplementary Data Table 8). All participants had received a two-dose primary course of COVID-19 vaccine approximately 6 months before study enrollment (Fig. 2a). As prior natural COVID-19 infection enhances subsequent vaccination responses, individuals with detectable anti-nucleocapsid antibodies (*n* = 2 with severe obesity; *n* = 1 normal BMI control) were excluded. Mean levels of anti-spike and anti-RBD IgG antibodies were similar between individuals with severe obesity and normal BMI 6 months after the second vaccine dose (Fig. 2b and Extended Data Fig. 3). By contrast, the function of these antibodies, measured by their ability to neutralize authentic SARS-CoV-2 viral infection (neutralizing titers at 50% inhibition (NT_50_))^30^, was reduced in individuals with severe obesity (Fig. 2c). Fifty-five percent of individuals with severe obesity had unquantifiable or undetectable neutralizing capacity compared to 12% of normal BMI controls (*Z* = 3.610, *P* = 0.0003, *Z*-test; Fig. 2d). Dissociation between anti-spike antibody levels and neutralizing capacity could be a consequence of lower antibody affinity or differential antibody reactivity to non-neutralizing epitopes on the spike protein. Here, the similar levels of RBD-binding antibodies across both groups indicate adequate capacity for antibody production against neutralizing epitopes and suggest a lower affinity of SARS-CoV-2 antibodies in individuals with severe obesity. Consistent with this observation, despite equivalent levels of anti-RBD antibodies, neutralizing capacity tended to be higher in individuals with a normal BMI (Extended Data Fig. 4). Baseline plasma glucose, leptin levels, diagnosis of type 2 diabetes or type of vaccine did not correlate with neutralizing capacity in individuals with severe obesity (Extended Data Fig. 3b–f).

Suboptimal antibody responses might be enhanced by activating memory B cells, which can rapidly differentiate into antibody-producing plasma cells after booster immunization. However, we found that circulating antigen-experienced (IgD^−^) RBD-binding B cells were similar in both groups (*P* = 0.1448; Fig. 2e and Extended Data Fig. 3g,h). Antigen-specific T cell responses quantified by ELISpot were also similar in individuals with severe obesity and normal BMI controls (Extended Data Fig. 3i; *P* = 0.2243 for V3D0, where V3D0 denotes vaccine 3 day 0 (sampling time-point before third dose)).

### Response to booster vaccination

**Fig. 3:**
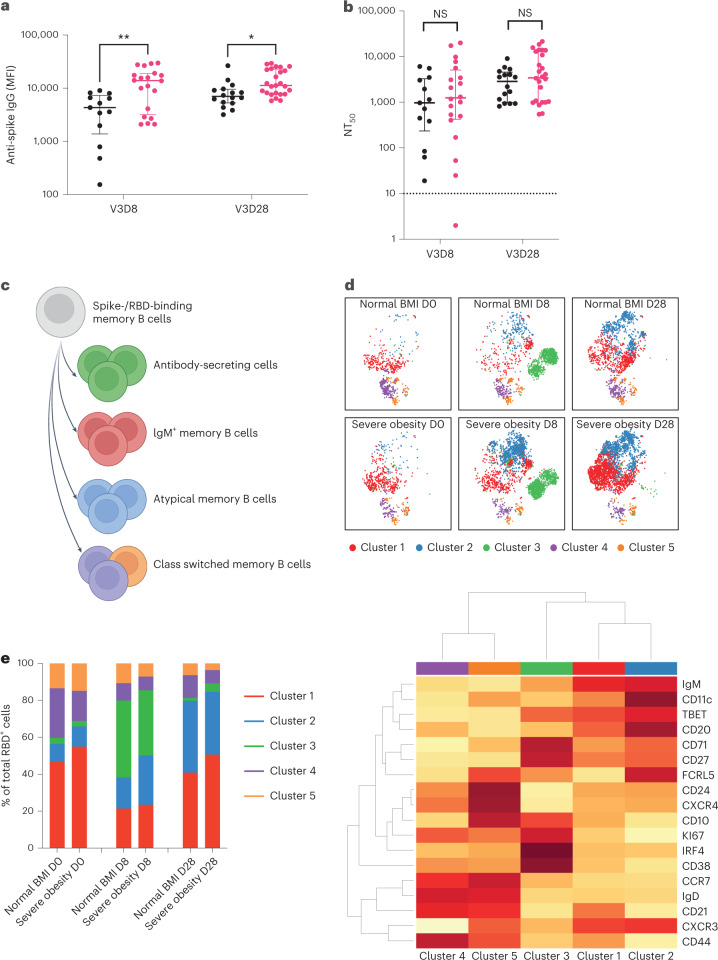
Immune response to third (booster) dose COVID-19 vaccination. Individuals with severe obesity (*n* = 25, magenta) and normal BMI controls (*n* = 16, black) were studied at day 8 (D8) and day 28 (D28) after the third vaccine dose (V3). **a**, Levels of anti-spike IgG antibodies (MFI) at day 8 (**P = 0.0024, mixed-effects analysis with Sidak’s multiple comparisons tests) and day 28 (*P = 0.0199, mixed-effects analysis with Sidak’s multiple comparisons tests). Horizontal bars indicate the median and interquartile range. **b**, NT_50_ against wild-type SARS-CoV-2 at days 8 and 28, with the dotted line indicating the limit of quantification. Horizontal bars indicate the median and interquartile range. **c**, Schematic depicting the differentiation of B cells in response to vaccine administration. **d**, High-dimensional spectral flow cytometry of SARS-CoV-2 RBD-binding B cells. tSNE and FlowSOM analyses of multi-parameter flow cytometry of CD19^+^ RBD-binding B cells from individuals with normal weight and individuals with severe obesity before (V3D0), 8 d after (V3D8) and 28 d after (V3D28) a booster mRNA vaccine (top panel). Bottom panel: heat map of row-normalized mean protein expression on different clusters of cells; red indicates high expression, and yellow indicates low expression. **e**, Frequency of RBD-binding B cells between groups over time. NS, not significant. Source data

**Extended Data Fig. 5:**
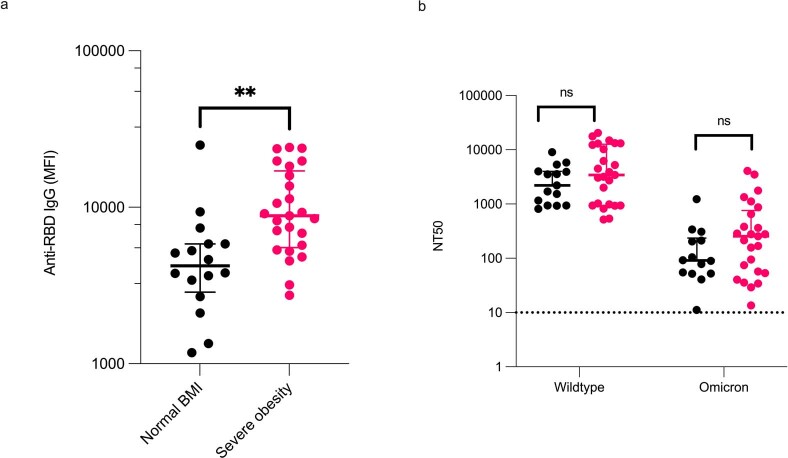
Response to third dose booster vaccination in severe obesity. a, Anti-RBD (Receptor Binding Domain) IgG levels (MFI, mean fluorescence intensity) were higher in people with severe obesity (magenta, n = 25) compared to individuals with normal BMI (black, n = 16) (**P = 0.0013 in Mann-Whitney U test). Each symbol represents an individual person studied at Day 28; line indicates the median. b, Neutralizing antibody titres (NT50) against the Omicron variant of SARS-CoV-2 were markedly reduced compared with wild type virus (Day 28), but no difference was observed between groups (severe obesity (magenta, n = 15) compared to normal BMI controls (n = 25, black)). Each symbol represents an individual person, horizontal bar indicates the median and interquartile range and p-values are from a Mann-Whitney U test. ns, not significant. Source data

We next studied the response to a third (booster) dose of mRNA vaccine (BNT162b2 or mRNA1273) in individuals with severe obesity (*n* = 28) and individuals with normal BMI (*n* = 16). As expected, levels of anti-spike and anti-RBD IgG antibodies increased markedly at day 28 (Fig. 3a and Extended Data Fig. 5). Peak levels were higher in individuals with severe obesity than in individuals with normal BMI (*P* = 0.0052, Fig. 3a and *P* = 0.0014, Extended Data Fig. 5a). Unlike anti-spike and anti-RBD antibody levels, neutralizing antibody titers were similar between the two groups at day 28 (Fig. 3b, *P* = 0.3534, and Extended Data Fig. 5b). Similar to the primary vaccine response, despite equivalent levels of anti-RBD antibodies, neutralizing capacity was higher in individuals with normal BMI (Extended Data Fig. 4b–f). Nonetheless, all individuals generated an NT_50_ > 100, and 61% (*n* = 17) of individuals with severe obesity and 67% (*n* = 8) of individuals with normal BMI generated an NT_50_ > 1,000.

**Extended Data Fig. 6:**
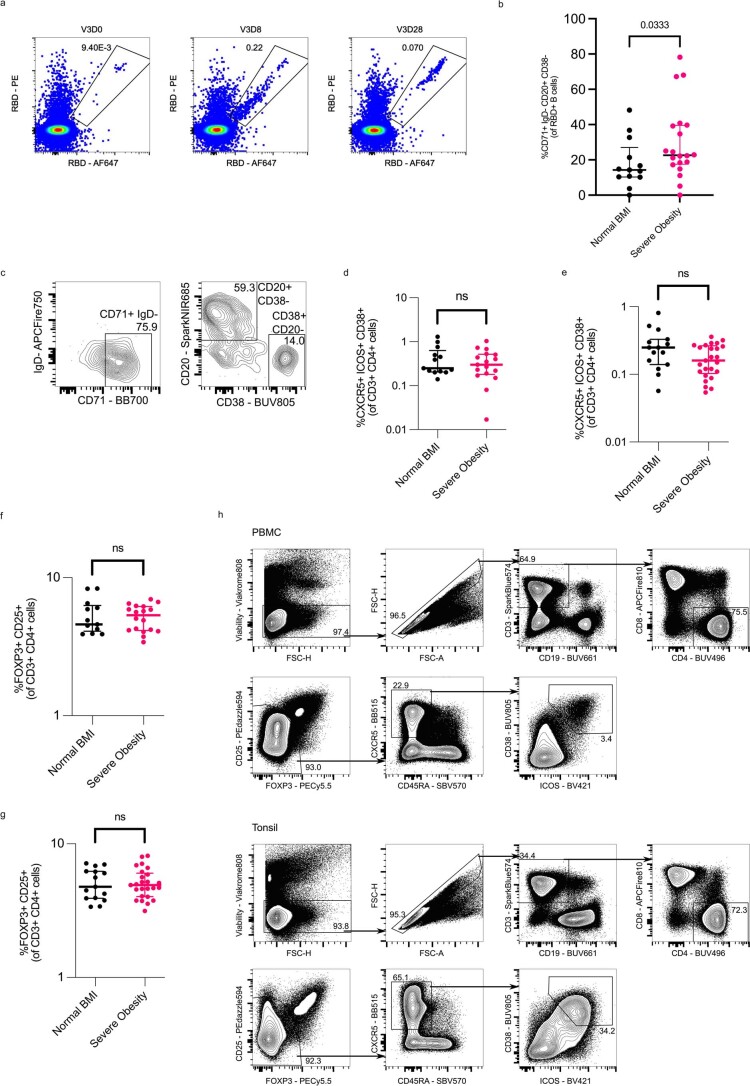
B and T cell response to third dose booster vaccination in severe obesity. **a**, Representative high dimensional spectral flow cytometry analysis in participant with severe obesity. Flow cytometric plots of RBD-binding in a patient with severe obesity, prior to (V3D0), eight days after (V3D8) and 28 days after (V3D28) a booster mRNA vaccine. **b**, Flow cytometric quantification of RBD-binding B cells of activated phenotype in normal BMI individuals (black, n = 20) and participants with severe obesity (magenta, n = 13) 8 days after (V3D8) a third dose mRNA vaccine. Each symbol represents an individual person, horizontal bars indicate the median and interquartile range and p-value is from a Mann-Whitney U test. *P = 0.0333 **c**, Representative flow cytometric plot-CD71 + IgD CD20 + CD38- gating strategy for S4b. **d-e**, Flow cytometric quantification of circulating T follicular helper (cTfh) cells (CXCR5 + ICOS + CD38 + FOXP3- CD25- CD4 + cells) in normal BMI individuals (black, n = 13 V3D8, n = 15 V3D28) and participants with severe obesity (magenta, n = 17 V3D8, n = 25 V3D28) 8 days (**d**) and 28 days (**e**) after a third dose mRNA vaccine. Each symbol represents an individual person, horizontal bars indicate the median and p-value is from a Mann-Whitney U test. **f-g**, Flow cytometric quantification of circulating T regulatory cells (FOXP3 + CD25 + CD4 + cells) in normal BMI individuals (black, n = 13 V3D8, n = 15 V3D28) and participants with severe obesity (magenta n = 18 V3D8, n = 25 V3D28) 8 days (**f**) and 28 days (**g**) after a third dose mRNA vaccine. **h**, Representative flow cytometry T cell gating, pre-gated on live, single, lymphocytes. Circulating TFH are gated as (CD19- CD20- CD3 + CD4 + FOXP3- CD25- CXCR5 + ICOS + CD38 + ) and T regulatory cells are gated as (CD19- CD20- CD3 + CD4 + FOXP3 + CD25 + ). Tonsil staining was performed alongside PBMC samples to identify bona fide Tfh cells. Each symbol represents an individual person, horizontal bars indicate the median and interquartile range and p-value is from a Mann-Whitney U test. Source data

To further assess humoral immunity associated with severe obesity, we next used an established high-dimensional spectral flow cytometry panel^31^ to enumerate and phenotype SARS-CoV-2 RBD-binding B cells (Fig. 3c–e and Extended Data Fig. 6a–c). Unsupervised t-distributed stochastic neighbor embedding (tSNE) analysis of RBD-binding (RBD^+^) cells was performed on all IgD^−^ B cells combined from individuals with normal BMI and individuals with severe obesity at several timepoints. Cluster 1 represented IgM^+^ B cells; cluster 2 showed features of atypical or age-associated B cells^32^; cluster 3 separated plasmablasts; and the smallest clusters (4 and 5) were class-switched memory B cells (Fig. 3c–e). Cells from individuals with severe obesity and from individuals with normal BMI were distributed within all clusters, at all timepoints, suggesting that obesity was not associated with a loss of B cell subset differentiation. However, classical bi-axial gating identified that individuals with severe obesity had an increased frequency of IgD^−^CD71^+^ RBD-binding B cells 8 d after the booster (Extended Data Fig. 6b,c). The number of circulating T follicular helper (cTfh) cells, a circulating biomarker of the germinal center reaction^33^, did not differ between the groups (Extended Data Fig. 6d–h). Consistent with this, antigen-specific T cell responses quantified by ELISpot and the number of regulatory T cells were similar in individuals with severe obesity and individuals with normal BMI (Extended Data Fig. 3i; *P* = 0.8173 for V3D8 and *P* = 0.8903 for V3D28).

### Waning of humoral immunity after booster vaccination

**Fig. 4:**
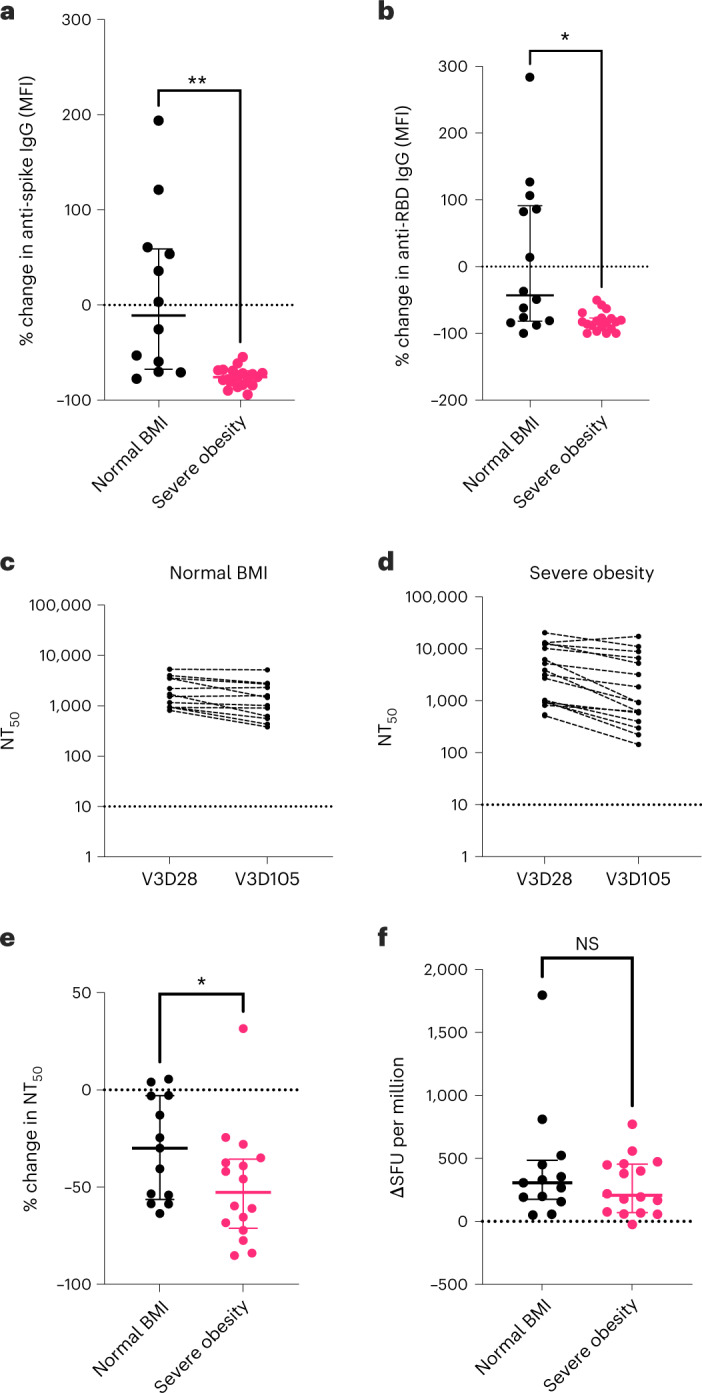
Third-dose COVID-19 vaccine-induced immunity in individuals with severe obesity. Individuals with severe obesity (*n* = 19, magenta) and normal BMI controls (*n* = 14, black) were studied at day 28 and day 105 after the third vaccine dose. **a**, Percentage (%) change in anti-spike IgG antibody levels (MFI). Horizontal bars indicate the median and interquartile range (**P = 0.0057 in Welch’s *t*-test). **b**, Percentage (%) change in anti-RBD IgG antibody levels between these two timepoints. Horizontal bars indicate the median and interquartile range (*P = 0.0102 in Welch’s *t*-test). **c**,**d**, NT_50_ measured at 28 d (V3D28) and 105 d (V3D105) after third-dose vaccination (V3) in normal BMI controls (**c**) and individuals with severe obesity (**d**). **e**, Percentage (%) change in NT_50_ against wild-type SARS-CoV-2, with the dotted line indicating the limit of quantification. Horizontal bars indicate the median and interquartile range (*P = 0.0448 in Mann–Whitney test). Dotted line indicates no change. **f**, T cell responses quantified by ELISpot. Horizontal bars indicate the median and interquartile range. Individuals who reported a positive SARS-CoV-2 RT–PCR test between day 28 and day 105 or who had positive anti-nucleocapsid antibodies at day 105 were excluded from these analyses. NS, not significant. Source data

**Extended Data Fig. 7:**
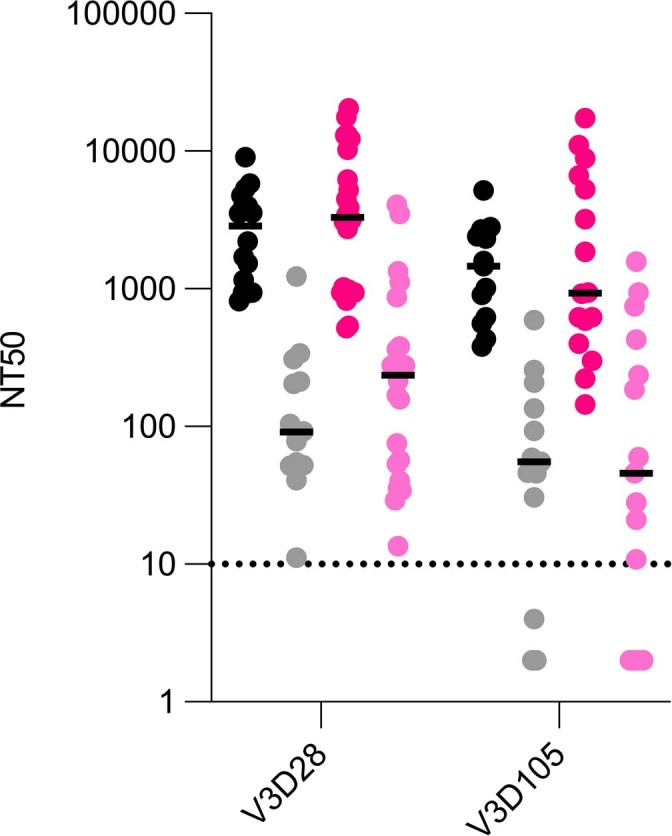
Neutralizing capacity against wildtype and Omicron (BA.1) variant SARS-CoV-2 at day 28 and day 105 post-third dose booster vaccination. NT50s against wildtype (black and magenta symbols) and Omicron (BA.1) variant SARS-CoV-2 (grey and pink) at day 28 and day 105 post-third dose booster vaccination in normal BMI individuals (black and grey, n = 15) and participants with severe obesity (n = 22, magenta and pink). Each symbol represents an individual person, horizontal bars indicate medians. Dotted line, limit of quantification. Source data

The lower neutralizing antibody titers observed in individuals with severe obesity before booster vaccination could reflect a reduction in either the peak response to primary vaccination or its longevity. We, therefore, measured antibody levels at day 28 and day 105 (15 weeks) after the third dose of vaccine. We found more rapid waning of anti-spike and anti-RBD IgG levels and neutralizing antibody titers in individuals with severe obesity (*P* = 0.0057 for percentage change in anti-spike IgG; *P* = 0.0087 for percentage change in anti-RBD IgG; *P* = 0.0220 for percentage change in NT_50_; Fig. 4a–e). Neutralizing capacity against the Omicron variant of SARS-CoV-2 (BA.1) was similarly reduced with time (Extended Data Fig. 7). Conversely, antigen-specific T cell responses quantified by ELISpot remained similar in individuals with severe obesity and individuals with normal BMI at day 105 (Fig. 4f). Taken together, these data indicate that severe obesity leads to a failure in the maintenance of humoral immunity after COVID-19 vaccination, associated with an increased risk of severe COVID-19.

## Discussion

Obesity is an established risk factor for severe COVID-19 (ref. ^2^). In this study of over 0.5 million vaccinated individuals with obesity and more than 98,000 vaccinated individuals with severe obesity, we found that an increased BMI is associated with an increased risk of hospitalization and mortality from breakthrough infections. In parallel, we found evidence of reduced neutralizing antibody capacity 6 months after primary vaccination in individuals with severe obesity. These changes in antibody kinetics are associated with a dissociation between anti-RBD antibody levels and neutralizing capacity. A similar, relative reduction in neutralizing capacity was previously observed in patients with severe COVID-19 in other settings^34,35^ and might reflect lower antibody affinities. Consistent with the kinetics of the neutralizing antibody response in individuals with severe obesity, we show an increased risk of severe outcomes of COVID-19 even after COVID-19 vaccination in individuals with severe obesity, which increases with time after the primary vaccination course.

Our findings in individuals with severe obesity are consistent with previous studies showing that COVID-19 vaccines are immunogenic in individuals with obesity and individuals with a normal BMI^19–24^. In addition, our finding that peak antibody levels were in fact higher in individuals with severe obesity than in individuals with a normal BMI argues against vaccine delivery failure in individuals with obesity due to short needle length^25^ (longer needles are recommended for individuals with BMI > 40 kg/m^2^ in the UK) and indicates that a fixed rather than a weight-adjusted dosing schedule is appropriate for COVID-19 vaccination. Some of these studies suggested that the duration of vaccine-induced immunity could be reduced in individuals with obesity^20–22^. These studies all relied on measurements of immunity at a single timepoint and used different assays and/or endpoints (for example, self-reported home antibody tests^23^ or an assumption of effectiveness in those who tested negative for COVID-19 by RT–PCR^24^). Here, by prospectively measuring B cell and T cell responses as well as neutralizing capacity of antibodies to authentic virus in vaccinated individuals with severe obesity and normal BMI over time, we demonstrate that the waning of humoral immunity associated with COVID-19 vaccines^36^ is accelerated in individuals with severe obesity. Furthermore, we demonstrate that severe obesity does not lead to a failure to target neutralizing spike epitopes. Rather, the relative reduction in neutralizing capacity may result from a lack of high-affinity antibodies. Indeed, our findings are similar to those seen in individuals with obesity after influenza vaccination, with no difference in early immunogenicity but quicker waning associated with higher risk of influenza or influenza-like illness in individuals with obesity throughout the influenza season^37^. Further studies will be needed to test whether hyperglycemia modifies the risk associated with severe obesity.

Limitations of the current study include the definition of severe COVID-19 outcomes in the EAVE II population study, which may include individuals who were hospitalized for other reasons but in whom infection was incidentally detected. This definition likely affects individuals in all BMI categories similarly (post hoc sensitivity analysis in clinically confirmed cases generated very similar results). BMI data were collected only once in EAVE II, and, thus, we cannot account for changes in BMI over time. However, there is substantial evidence that the secular trend is for BMI to increase over time, which means that any bias is likely to operate differentially toward the null (some individuals classified as having normal BMI might have obesity). Any misclassification error is, therefore, likely to underestimate associations between high BMI and severe COVID-19 outcomes. The number of individuals studied in the SCORPIO study was relatively modest and means that there is a risk of type II errors. Due to sample size, it is not feasible to perform a multivariate analysis to account for other variables, which could potentially affect immunogenicity. Additionally, our study is underpowered to directly test whether there is an association between type 2 diabetes and/or drugs prescribed for metabolic disease and specific immuno-phenotypes.

There is substantial evidence that weight loss of at least 5% can reduce the risk of type 2 diabetes and other metabolic complications. Accordingly, it is likely that lifestyle modification, pharmacotherapy and bariatric surgery—interventions that can lead to a reduction in body weight and improvement in metabolic health—could similarly ameliorate COVID-19 outcomes. Further studies in individuals in the weight-reduced state will be important to establish whether weight loss can mediate beneficial effects on humoral immunity and, thus, offer improved protection against COVID-19 in both vaccinated and unvaccinated individuals. We conclude that individuals with obesity show a reduction in the maintenance of humoral vaccine responses, and we suggest that additional or more frequent booster doses are likely to be required to maintain protection against COVID-19. Due to the high prevalence of obesity^38^, this poses a major challenge for health services and vaccine programs around the world.

## Methods

### EAVE II study

The EAVE II surveillance platform drew on near-real-time nationwide healthcare data for 5.4 million individuals (~99%) in Scotland^26–29^. It includes information on clinical and demographic characteristics of each individual, their vaccination status and type of vaccine used and information on positive SARS-COV-2 infection and subsequent hospitalization or death. Ethical approval was granted by the National Research Ethics Service Committee, Southeast Scotland 02 (reference 12/SS/0201) for the study using the EAVE II platform. Approval for data linkage was granted by the Public Benefit and Privacy Panel for Health and Social Care (reference 1920-0279). Individual written patient consent was not required for this analysis.

Using data from the EAVE II platform, we examined the impact of obesity (using BMI measurements) and clinical and demographic characteristics, including time since receiving the second and third vaccine doses, previous history of testing positive for COVID-19, gap between vaccine doses and dominant variant in the background, of fully vaccinated adults in Scotland who experienced severe COVID-19 outcomes. The cohort analyzed for this study consisted of individuals aged 18 years and older who were administered with at least two doses of BNT162b2 mRNA, ChAdOx1 nCoV-19 or mRNA-1273 vaccines between 8 December 2020 and 19 March 2022. Follow-up began 14 d after receiving the second dose until COVID-19-related hospitalization, COVID-19-related death or the end of study period (that is, 19 March 2022). All the COVID-19-related hospital admissions or deaths were selected between 14 September 2021 and 19 March 2022. We excluded events that occurred within the first 14 d after completing the primary vaccination schedule to allow for time for a full immune response to be mounted. Patients without immunosuppression had their primary vaccination schedule with two doses, and so the third dose is a booster. For people with immunosuppression, the primary vaccination schedule was for three vaccine doses. BMI was available for individuals based on last recorded measurement within their primary care record. Where the BMI was missing, it was imputed using ordinary least squares regression with all QCovid risk groups^39^ together with age, sex and deprivation included as predictors. The coding systems used in Scotland are Read for GP data and ICD-10 for hospitalization data. This information is provided in more detail in the EAVE II protocol and cohort profile^39^ and data dictionary (https://www.ed.ac.uk/usher/eave-ii) and at https://github.com/EAVE-II/EAVE-II-data-dictionary.

#### Definition of outcomes

The primary outcome of interest was severe COVID-19, which was defined as COVID-19-related hospital admission or death 14 d or more after receiving the second vaccine or booster dose^40^. COVID-19-related hospital admission was defined as hospital admission within 14 d of a positive RT–PCR test or COVID-19 as reason for admission or a positive SARS-CoV-2 RT–PCR test result during an admission where COVID-19 was not the reason for admission. COVID-19-related mortality was defined as either death for any reason within 28 d of a positive RT–PCR test or where COVID-19 was recorded as the primary reason for death on the death certificate.

#### Population characteristics and confounders

Characteristics of interest were defined at baseline on 8 December 2020 and included age, sex, socioeconomic status based on quintiles of the SIMD, urban or rural place of residence (which is a measure of rurality based on residential settlement), BMI, previous natural infection from SARS-CoV-2 before second dose of the vaccine (classified as 0–3 months, 3–6 months, 6–9 months and ≥9 months before second vaccine dose), number of pre-existing comorbidities known to be linked with severe COVID-19 outcome and being in a high-risk occupational group, defined as someone with undergoing regular RT–PCR testing^27^. Time since vaccination was distributed in the periods of 3–10 weeks, 11–15 weeks, 16–20 weeks and ≥21 weeks from completion of second dose of the primary vaccination and 3–5 weeks, 6–8 weeks and ≥9 weeks for the booster doses separately. To allow for variation in background levels of community infection, we split the data by calendar week. BMI was grouped as <18.5 kg/m^2^ (underweight), 18.5–24.9 kg/m^2^ (normal weight), 25–29.9 kg/m^2^ (overweight), 30–39.9 kg/m^2^ (obese) and ≥40 kg/m^2^ (severely obese) according to World Health Organization (WHO) criteria.

#### EAVE II statistical analysis

We calculated the frequency and rate per 1,000 person-years of severe COVID-19 outcomes for all demographic and clinical factors. Generalized linear models (GLMs) assuming a Poisson distribution with person-time as an offset representing the time at risk were used to derive rate ratios (RRs) with 95% CIs for the association between demographic and clinical factors and COVID-19-related hospitalization or death. aRRs were estimated adjusting for all confounders, including age, sex, SIMD, time since receiving the second dose of vaccine, pre-existing comorbidities, the gap between vaccine doses, previous history of SARS-CoV-2 infection and calendar time. SIMD looks at the extent to which an area is deprived across seven domains: income, employment, education, health, access to services, crime and housing. SIMD was allocated based on an individual’s home postcode, with quintiles of population ranging from 1 for the most deprived 20% to 5 for the least deprived 20% of the population.

We conducted a post hoc sensitivity analysis in clinically confirmed cases. Because BMI was not available for all the individuals, missing BMI was imputed using ordinary least squares regression with all other independent variables included as predictors. We conducted a post hoc sensitivity analysis by imputing the missing BMI using an average of 10 least squares regressions (multiple imputation). R (version 3.6.1) was used to carry out all statistical analyses.

### Ethical approval and study populations

#### SCORPIO clinical study

Clinical studies in individuals with severe obesity and normal BMI controls were approved by the National Research Ethics Committee and the Health Research Authority (East of England–Cambridge Research Ethics Committee (SCORPIO study amendment of ‘NIHR BioResource’ 17/EE/0025)). Human tonsil samples were collected under ethical approval from the UK Health Research Authority (reference 16/LO/0453). Each participant provided written informed consent. All studies were conducted in accordance with the Declaration of Helsinki and the guidelines for Good Clinical Practice.

Individuals with severe obesity (class II/III WHO criteria of BMI ≥ 40 kg/m^2^ or BMI ≥ 35 kg/m^2^ with obesity-associated medical conditions, such as type 2 diabetes and hypertension) who attended the obesity clinic at Cambridge University Hospitals NHS Trust and had received two doses of COVID-19 vaccination (first and second doses of ChAdOX1 nCoV-19 or BNT162b2 mRNA) between December 2021 and May 2022 were invited to take part. Individuals with acquired (HIV, immunosuppressant drugs) or congenital immune deficiencies and cancer were excluded. Third-dose vaccinations (BNT162b2, Pfizer BioNTech or half-dose mRNA1273 (Moderna)) were administered as part of the NHS vaccination program. UK Health Security Agency policy recommends the use of longer needles (38 mm versus 25 mm) in individuals with severe obesity.

Additional normal BMI controls were recruited in Oxford, UK, as part of the PITCH study under the GI Biobank Study 16/YH/0247, approved by the Yorkshire & Humber Sheffield Research Ethics Committee, which was amended for this purpose on 8 June 2020. Samples obtained 6 months after the primary course were included.

Clinical and immunological measurements were taken before the booster vaccination and 8 d (−3), 28 d (±7) and 105 d (±7) after vaccination. Third-dose vaccinations were administered as part of the NHS vaccination program and were mRNA vaccines (BNT162b2 or mRNA1273 (Moderna)). Clinical data regarding comorbidities associated with obesity were obtained from the medical records. Supplementary Data Table 8 details the demographic characteristics of this cohort. Healthy healthcare workers were enrolled into the longitudinal OPTIC cohort in Oxford, UK, between May 2020 and May 2021 as part of the PITCH consortium. PITCH participants were sampled between July and November 2021, a median of 185 d (range, 155–223) after receiving a second vaccination with ChAdOX1 nCoV-19 or BNT162b2 mRNA vaccine. All PITCH participants were classified as infection-naive, as defined by never having received a positive lateral flow or PCR test for SARS-CoV-2 and negative anti-nucleocapsid antibodies at the time of their first vaccination. Therefore, a total of 28 individuals with severe obesity and 41 normal BMI controls were evaluated 6 months after the primary course of vaccination, whereas, for the response to third-dose vaccination, 16 normal BMI controls were studied.

Of the 28 recruited individuals with severe obesity, two had positive anti-nucleocapsid antibodies and reported a positive RT–PCR test before their third-dose vaccination. They were excluded from further analysis. In addition, between day 28 and day 105, two individuals with severe obesity reported positive SARS-CoV-2-tests (lateral flow test or RT–PCR tests as per UK guidelines at the time). They were excluded from the day 28 to day 105 analysis. An additional three people with severe obesity were recruited after they had had their booster for day 28 and day 105 analysis only. In addition, one of the normal-weight individuals had positive anti-nucleocapsid antibodies who had not had a PCR test, before their third-dose vaccination. This individual was excluded from the before and after third-dose analysis. In addition, between day 28 and day 105, two normal-weight individuals reported positive SARS-CoV-2-tests (lateral flow test or PCR tests as per UK guidelines at the time, one of those individuals on two separate occasions). They were excluded from the day 28 to day 105 analysis. Missing data in addition to this were due to (1) occasional difficult venepuncture in individuals with obesity; (2) insufficient peripheral blood mononuclear cells (PBMCs) isolated for both T and B cell analysis; or (3) insufficient sample to run both wild-type and Omicron neutralization assays. Therefore, we specified how many individuals were included per analysis, per figure.

Peripheral blood samples were acquired in either lithium heparin (PBMCs) or serum-separating tubes. Serum tubes were centrifuged at 1,600*g* for 10 min at room temperature to separate serum from the cell pellet before being aliquoted and stored at −80 °C until use. PBMCs were isolated by layering over Lymphoprep density gradient medium (STEMCELL Technologies), followed by density gradient centrifugation at 800*g* for 20 min at room temperature. PBMCs were isolated and washed twice using wash buffer (1× PBS, 1% FCS, 2 mM EDTA) at 400*g* for 10 min at 4 °C. Isolated PBMCs were resuspended in freezing media, aliquoted and stored at −80 °C for up to 1 week before being transferred to liquid nitrogen until use.

#### SARS-CoV-2 serology by multiplex particle-based flow cytometry

Recombinant SARS-CoV-2 nucleocapsid, spike and RBD were covalently coupled to distinct bead sets (Luminex) to form a three-plex and analyzed as previously described^41^. Specific binding was reported as mean fluorescence intensity (MFI).

#### Neutralizing antibodies to SARS-CoV-2

Luminescent HEK293T-ACE2-30F-PLP2 reporter cells (clone B7) expressing ACE2 and SARS-CoV-2 papain-like protease-activatable circularly permuted firefly luciferase (FFluc) are available from the National Institute for Biological Standards and Control (NIBSC, https://www.nibsc.org/, cat. no. 101062)^30^. They were cultured in IMDM supplemented with 4 mM GlutaMAX (Gibco), 10% FCS, 100 U ml^−1^ penicillin and 0.1 mg ml^−1^ streptomycin at 37 °C in 5% CO_2_, regularly screened and confirmed to be mycoplasma negative (Lonza MycoAlert).

The SARS-CoV-2 viruses used in this study were a wild-type (lineage B) isolate (SARS-CoV-2/human/Liverpool/REMRQ0001/2020), a kind gift from Ian Goodfellow (University of Cambridge), isolated by Lance Turtle (University of Liverpool), David Matthews and Andrew Davidson (University of Bristol)^42,43^, and an Omicron (lineage B.1.1.529) variant, a kind gift from Ravindra Gupta^44^. Unless otherwise indicated, all data shown refer to neutralization of wild-type virus.

Sera were heat inactivated at 56 °C for 30 min before use, and NT_50_ values were measured as previously described^30,45^. In brief, luminescent HEK293T-ACE2-30F-PLP2 reporter cells (clone B7) expressing SARS-CoV-2 papain-like protease-activatable circularly permuted FFluc were seeded in flat-bottomed 96-well plates. The next day, SARS-CoV-2 viral stock (multiplicity of infection (MOI) = 0.01) was pre-incubated with a three-fold dilution series of each serum for 2 h at 37 °C and then added to the cells. Sixteen hours after infection, cells were lysed in Bright-Glo Luciferase Buffer (Promega) diluted 1:1 with PBS and 1% NP-40, and FFluc activity was measured by luminometry.

Experiments were conducted in duplicate. To obtain NT_50_ values, titration curves were plotted as FFluc versus log (serum dilution) and then analyzed by nonlinear regression using the Sigmoidal, 4PL, X is log(concentration) function in GraphPad Prism. NT_50_ values were reported when (1) at least 50% inhibition was observed at the lowest serum dilution tested (1:10) and (2) a sigmoidal curve with a good fit was generated. For purposes of visualization and ranking, samples with no neutralizing activity were assigned an arbitrary NT_50_ value of 2. Samples for which visual inspection of the titration curve indicated inhibition at low dilutions, but that did not meet criteria (1) and (2) above, were assigned an arbitrary NT_50_ value of 4.

**Extended Data Fig. 8:**
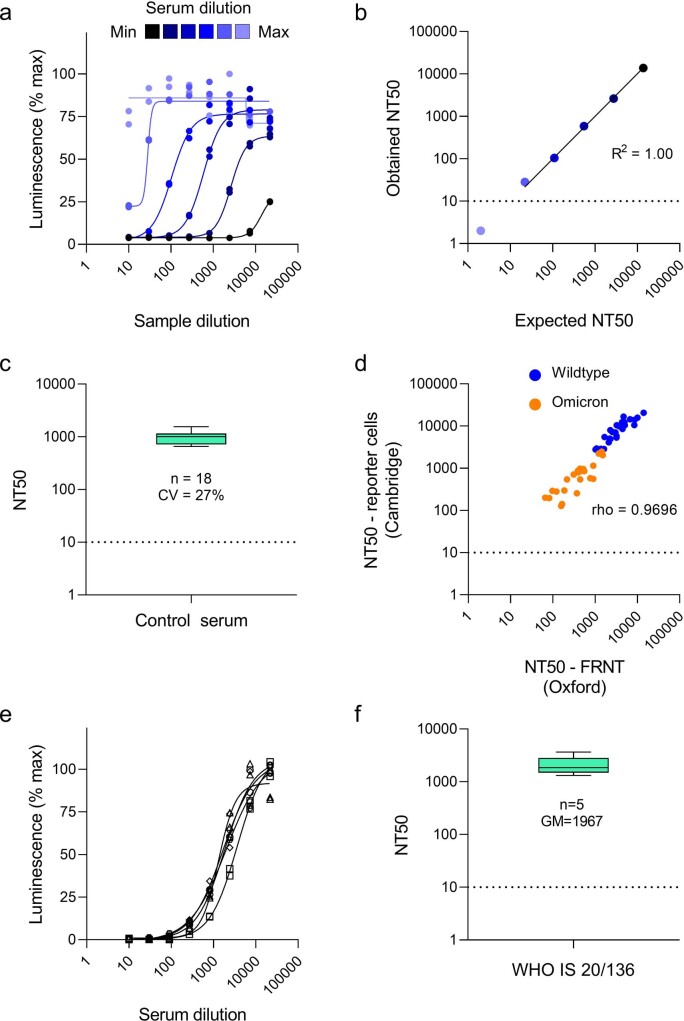
Quality assurance and calibration of assays for neutralizing antibodies to SARS-CoV-2. **a-b**, Linearity of the assay. Neutralization curves (**a**) and corresponding expected/obtained NT50s (**b**) against wildtype SARS-CoV-2 for a dilution series of high titre positive control serum. Infection of reporter cells is quantified as % maximum luminescence at 16 h. Mean values ± SEM are shown for experiments performed in duplicate (**a**). Solid line, line of best fit (dilutions above the limit of quantification). R2, coefficient of determination (dilutions above the limit of quantification). Dotted line, limit of quantification. **c**, Precision of the assay. NT50s against wildtype SARS-CoV-2 for a medium titre positive control serum sample from 18 independent experiments are summarised as a Tukey boxplot. CV, inter-assay coefficient of variation. Dotted line, limit of quantification. **d**, External validation of the assay. Comparison of NT50s against both wildtype (blue) and Omicron (BA.1) variant (orange) SARS-CoV-2 from this paper (reporter cells, Cambridge) with results of Focus Reduction Neutralization Tests (FRNTs, Oxford) for a panel of 28 serum samples from convalescent plasma donors. rho, Spearman’s rank correlation coefficient. Dotted line, limit of quantification. **e-f**, Calibration of the assay. Neutralization curves (**e**) and corresponding NT50s (**f**) against wildtype SARS-CoV-2 for WHO International Standard 20/136 (WHO IS 20/136) from 5 independent experiments. Infection of reporter cells is quantified as % maximum luminescence at 16 h. Mean values ± SEM are shown for experiments performed in duplicate (**e**). NT50s are summarised as a Tukey boxplot (e Minimum: 654, Maximum: 1550, Median: 1006, 25% percentile: 718. 75% percentile: 1157 and f Minimum: 1315, Maximum: 3634, Median: 1851, 25% percentile: 1485, 75% percentile: 2822, Geometric Mean: 1967). GM, geometric mean. Dotted line, limit of quantification. Source data

To confirm the linearity of the assay, a high-titer positive control serum sample was spiked into FCS (serum dilution series), and then each dilution was treated as a separate sample. Expected and obtained NT_50_ values against wild-type SARS-CoV-2 were compared by linear regression, generating a coefficient of determination (R^2^) of 1.00 for dilutions above the limit of quantification (Extended Data Fig. 8a,b).

As a measure of intermediate precision^46^, inter-assay variability was quantified for a medium-titer control serum sample tested against wild-type SARS-CoV-2 in 18 independent experiments conducted over a period 18 months by two different laboratory scientists, revealing a coefficient of variation (CV) of 27% (Extended Data Fig. 8c).

For external validation, a panel of 28 serum samples from NHS Blood and Transplant convalescent plasma donors participating in the C-VELVET study (approved by the West Midlands Solihull Research Ethics Committee, reference 21/WM/0082, IRAS project ID 296926) was tested blinded against both wild-type and Omicron variant SARS-CoV-2. NT_50_ values were compared with previously reported focus reduction neutralization test (FRNT) results obtained at the University of Oxford^47^, revealing a Spearman’s rank correlation coefficient (rho) of 0.9696 (Extended Data Fig. 8d).

Finally, to enable comparison with other studies, the neutralizing capacity of WHO International Standard 20/136 against wild-type SARS-CoV-2 was measured in five independent experiments, yielding a geometric mean NT_50_ of 1,967 (Extended Data Fig. 8e,f). This standard comprises pooled convalescent plasma obtained from 11 individuals which, when reconstituted, is assigned an arbitrary neutralizing capacity of 1,000 IU ml^−1^ against early 2020 SARS-CoV-2 isolates^48^. NT_50_ values against wild-type SARS-CoV-2 from this study may, therefore, be converted to IU ml^−1^ using a calibration factor of 1,000/1,967 (0.51), with a limit of quantification of 5.1 IU ml^−1^ (corresponding to an NT_50_ value of 10).

#### T cell cytokine production

Antigen-specific T cell responses were assessed using an ELISpot assay as previously described^49^. Results are expressed as spot forming units (SFU) per 10^6^ PBMCs. Analysis was completed using GraphPad Prism software version 9.3.1. The comparison of means between groups was performed using two-way, mixed-model ANOVA.

#### Quantitation of lymphocyte types and subsets by spectral flow cytometry

Generation of RBD-specific B cell probes and measurement of RBD-specific B cells were measured by high-dimension flow cytometry as described previously^50,51^. In brief, for flow cytometry stains, a single-cell suspension was prepared from cryopreserved PBMC samples as follows. First, 1 ml of PBMC samples was de-frosted in a 37 °C water bath and then immediately diluted into 9 ml of pre-warmed RPMI + 10% FBS. Cells were washed twice with 10 ml of FACS buffer (PBS containing 2% FBS and 1 mM EDTA). Cells were then resuspended in 500 µl of FACS buffer, and cell numbers and viability were determined using a Countess automated cell counter (Invitrogen). Next, 5 × 10^6^ viable cells were transferred to 96-well plates for antibody staining (dilutions used are provided in Supplementary Table 9). Cells were then washed once with FACS buffer and stained with 100 µl of surface antibody mix (including B cell probes) for 2 h at 4 °C. Cells were then washed twice with FACS buffer and fixed with the eBioscience Foxp3/Transcription Factor Staining Buffer (Thermo Fisher Scientific, 00-5323-00) for 30 min at 4 °C. Cells were then washed with 1× eBioscience Foxp3/Transcription Factor Permeabilization Buffer (Thermo Fisher Scientific, 00-8333-56) twice and stained with intracellular antibody mix in permeabilization buffer at 4 °C overnight. After overnight staining, samples were washed twice with 1× permeabilization buffer and once with FACS buffer and acquired on a Cytek Aurora. Cells for single-color controls were prepared in the same manner as the fully stained samples. Manual gating of flow cytometry data was performed using FlowJo version 10.8 software (Tree Star).

#### SCORPIO study statistical analysis

Analysis was completed using GraphPad Prism software version 9.3.1. The comparison of means or medians between groups was performed using two-sided parametric *t*-tests, non-parametric Mann–Whitney *U*-tests or mixed-model tests when appropriate. tSNE, FlowSOM and heat map analyses were performed using R (version 4.1.2), using code that was previously described^52^.

### Reporting summary

Further information on research design is available in the Nature Portfolio Reporting Summary linked to this article.

## Online content

Any methods, additional references, Nature Portfolio reporting summaries, source data, extended data, supplementary information, acknowledgements, peer review information; details of author contributions and competing interests; and statements of data and code availability are available at 10.1038/s41591-023-02343-2.

## Supplementary information

Supplementary Information Supplementary Data Tables 1–10.

Reporting Summary

## Data Availability

The epidemiology study data that support the findings of this study are not publicly available because they are based on de-identified national clinical records. These are, however, available by application via Scotland’s National Safe Haven from Public Health Scotland. The data used in this study can be accessed by researchers through NHS Scotland’s Public Benefit and Privacy Panel via its Electronic Data Research and Innovation Service. Anonymized data from the SCORPIO study are included in the manuscript; stored samples are available from the corresponding authors upon reasonable request. Source data are provided with this paper.

## Source data

Source Data Fig. 1 Statistical source data.
Source Data Fig. 2 Statistical source data.
Source Data Fig. 3 Statistical source data.
Source Data Fig. 4 Statistical source data.
Source Data Extended Data Fig. 1 Statistical source data.
Source Data Extended Data Fig. 3 Statistical source data.
Source Data Extended Data Fig. 4 Statistical source data.
Source Data Extended Data Fig. 5 Statistical source data.
Source Data Extended Data Fig. 6 Statistical source data.
Source Data Extended Data Fig. 7 Statistical source data.
Source Data Extended Data Fig. 8 Statistical source data.

## Code availability

All code used in the EAVE II study is publicly available at https://github.com/EAVE-II/covid-obesity-humoral-response.
