# Supplementary material for: Accelerated waning of the humoral response to COVID-19 vaccines in obesity
Source: Nat Med. 2023 May 11;29(5):1146–54. doi: 10.1038/s41591-023-02343-2 (PMC10202802; doi:10.1038/s41591-023-02343-2)
Supplement: Supplementary file 1 — Supplementary Information Supplementary Data Tables 1–10. [file 41591_2023_2343_MOESM1_ESM.pdf]

nature medicine

Article

https://doi.org/10.1038/s41591-023-02343-2

Accelerated waning of the humoral response to COVID-19 vaccines in obesity

In the format provided by the authors and unedited

Supplementary Data Table 1: Association between BMI and COVID-19 hospitalization or death among individuals from the EAVE II cohort

BMI category (kg/m^2)	Adjusted rate ratios (95% CI)	
	BMI (imputed)	BMI (recorded)
**18.5-24.9**	1.0	1.0
**<18.5**	1.28 (1.12-1.47)	1.47 (1.27-1.69)
**25-29.9**	0.91 (0.86-0.96)	0.92 (0.83-1.01)
**30-39.9**	1.11 (1.05-1.18)	0.94 (0.84-1.04)
**40+**	1.76 (1.60-1.94)	1.36 (1.13-1.64)

Adjusted rate ratios (aRRs) were estimated adjusting for all confounders including age, sex, Scottish Index of Multiple Deprivation, time since receiving the second dose of vaccine, number of pre-existing comorbidities, the gap between vaccine doses, previous history of SARS-CoV-2 infection and calendar time. Where the BMI was missing, it was imputed using ordinary least squares regression with all other independent variables included as predictors (BMI (imputed)). CI, confidence intervals. BMI=body mass index.

Supplementary Data Table 2: Population characteristics of the EAVE II population above the age of 80 years old

**Characteristic**		**All individuals over 80 years of age**	
		**Total vaccination (n, %)**	**Severe COVID-19 outcome (n, rate per 1000 person-years)**
**Total**		225,947 (100.0)	2988 (26.8)
**BMI (kg/m^2)**	<18.5	5115 (2.3)	120 (44.9)
	18.5-24.9	52658 (23.3)	776 (30.5)
	25-29.9	124650 (55.2)	1479 (24.0)
	30-39.9	41132 (18.2)	579 (28.1)
	40+	2365 (1.0)	34 (32.3)

Supplementary Data Table 3: Sensitivity analysis (association between BMI and COVID-19 hospitalization or death among individuals from the EAVE II cohort) restricted to BMI imputed through multiple imputations method and clinically diagnosed severe COVID-19 outcomes

BMI category (kg/m^2)	Adjusted rate ratios (95% CI)	
	**Multiple imputed BMI**	**Clinically confirmed severe COVID-19 events**
**18.5-24.9**	1.0	1.0
**<18.5**	1.23 (1.09-1.39)	1.37 (1.16-1.62)
**25-29.9**	0.95 (0.89-1.03)	0.91 (0.85-0.98)
**30-39.9**	1.08 (1.00-1.16)	1.21 (1.12-1.30)
**40+**	1.62 (1.48-1.79)	2.09 (1.87-2.35)

Adjusted rate ratios (aRRs) were estimated adjusting for all confounders including age, sex, Scottish Index of Multiple Deprivation, time since receiving the second dose of vaccine, number of pre-existing comorbidities, the gap between vaccine doses, previous history of SARS-CoV-2 infection and calendar time. Where the BMI was missing, it was imputed using ordinary least squares regression with all other independent variables included as predictors (BMI (imputed)). CI, confidence intervals. BMI=body mass index.

Supplementary Data Table 4: Population characteristics of individuals from EAVE-II who received at least second (of the primary vaccination schedule) or third dose of a SARS-CoV-2 vaccine, and individuals who were unvaccinated during the study period.

		**EAVE II study cohort i.e. individuals who had received at least received second dose (of the primary vaccination schedule) or booster dose**		**Unvaccinated individuals in the Scottish population during the study period**	
**Characteristic**		**Total vaccination (n, %)**	**Severe COVID-19 outcome (n, rate per 1000 person-years)**	**Total vaccination (n, %)**	**Severe COVID-19 outcome (n, rate per 1000 person-years)**
**Total**		3,588,340 (100.0)	10,938 (6.0)	569,218 (100.0)	17,884 (31.4)
**BMI (kg/m²)**	<18.5	36,197 (1.0)	252 (13.7)	10,319 (1.8)	413 (40.0)
	18.5-24.9	456,128 (12.7)	1,813 (7.7)	76,289 (13.4)	2,765 (36.2)
	25-29.9	2,428,889 (67.7)	5,599 (4.5)	422,229 (74.2)	10,698 (25.3)
	30-39.9	568,420 (15.8)	2,710 (9.3)	52,135 (9.2)	3,374 (64.7)
	40+	98,706 (2.8)	609 (12.2)	8,246 (1.4)	634 (76.9)

Supplementary Data Table 5: Population characteristics of the EAVE II population, stratified by BMI groups

	<18.5 (number of individuals, number of events)	18.5-24.9 (number of individuals, number of events)	25.0-29.9 (number of individuals, number of events)	30.0-39.9 (number of individuals, number of events)	40+ (number of individuals, number of events)
**Sex**					
Female	26,187 (179)	303,678 (997)	1,163,267 (2,683)	318,072 (1,293)	68,374 (376)
Male	10,010 (73)	152,450 (816)	1,265,622 (2,916)	250,348 (1,417)	30,332 (233)
**Age groups (years)**					
18-49	18,753 (38)	217,886 (296)	1,188,635 (1,319)	170,045 (402)	39,105 (162)
50-64	5,811 (30)	93,801 (233)	692,170 (1,264)	192,959 (718)	36,611 (202)
65-79	6518 (64)	91,756 (508)	423,434 (1,537)	164,284 (1,011)	20,625 (211)
80+	5,115 (120)	52,685 (776)	124,650 (1,479)	41,132 (579)	2,365 (34)
**Deprivation status (SIMD)**					
1 - High	8,719 (81)	77,198 (450)	417,411 (1,503)	121,531 (791)	26,797 (244)
2	7,388 (54)	82,661 (420)	457,123 (1,282)	125,192 (694)	24,308 (147)
3	6,576 (40)	90,111 (335)	486,432 (1,068)	118,072 (524)	19,708 (79)
4	6,423 (41)	97,147 (298)	520,800 (934)	111,684 (420)	16,552 (77)
5 - Low	6,770 (33)	105,774 (297)	531,767 (787)	89,150 (270)	10,902 (60)
NA	330 (<10)	3,237 (13)	15,356 (25)	2,791 (<10)	439 (<10)
**Number of risk groups**					
0	15,816 (33)	229,528 (239)	1,549,890 (1,869)	208,753 (356)	33,671 (100)
1	10,128 (63)	129,770 (416)	610,365 (1,452)	187,235 (341)	33,825 (173)
2	5,362 (53)	54,626 (432)	178,034 (1,005)	97,780 (656)	18,404 (127)
3	2,766 (52)	24,203 (320)	56,525 (628)	43,786 (499)	7,866 (101)
4	1,280 (28)	10,773 (219)	21,447 (361)	18,899 (273)	3,056 (51)
5+	845 (23)	7,174 (187)	12,628 (284)	11,967 (285)	1,884 (57)

Supplementary Data Table 6: STROBE and RECORD checklists

	**Item No.**	**STROBE items**	**RECORD items**	**Location in the pdf manuscript where items are reported**
**Title and abstract**				
	1	(a) Indicate the study's design with a commonly used term in the title or the abstract<br>(b) Provide in the abstract an informative and balanced summary of what was done and what was found	RECORD 1.1: The type of data used should be specified in the title or abstract. When possible, the name of the databases used should be included.<br>RECORD 1.2: If applicable, the geographic region and timeframe within which the study took place should be reported in the title or abstract.<br>RECORD 1.3: If linkage between databases was conducted for the study, this should be clearly stated in the title or abstract.	p. 1
**Introduction**				
Background rationale	2	Explain the scientific background and rationale for the investigation being reported		p. 2
Objectives	3	State specific objectives, including any prespecified hypotheses		p. 2
**Methods**				
Study Design	4	Present key elements of study design early in the paper		P 2
Setting	5	Describe the setting, locations, and relevant dates, including periods of recruitment, exposure, follow-up, and data collection		p. 2
Participants	6	*(a) Cohort study* - Give the eligibility criteria, and the sources and methods of selection of participants. Describe methods of follow-up<br>*Case-control study* - Give the eligibility	RECORD 6.1: The methods of study population selection (such as codes or algorithms used to identify subjects) should be listed in detail. If this is not possible, an explanation should be provided.	p. 2-3

		criteria, and the sources and methods of case ascertainment and control selection. Give the rationale for the choice of cases and controls<br>*Cross-sectional study* - Give the eligibility criteria, and the sources and methods of selection of participants<br><br>*(b) Cohort study* - For matched studies, give matching criteria and number of exposed and unexposed<br>*Case-control study* - For matched studies, give matching criteria and the number of controls per case	RECORD 6.2: Any validation studies of the codes or algorithms used to select the population should be referenced. If validation was conducted for this study and not published elsewhere, detailed methods and results should be provided.<br><br>RECORD 6.3: If the study involved linkage of databases, consider use of a flow diagram or other graphical display to demonstrate the data linkage process, including the number of individuals with linked data at each stage.	
Variables	7	Clearly define all outcomes, exposures, predictors, potential confounders, and effect modifiers. Give diagnostic criteria, if applicable.	RECORD 7.1: A complete list of codes and algorithms used to classify exposures, outcomes, confounders, and effect modifiers should be provided. If these cannot be reported, an explanation should be provided.	p.10
Data sources/ measurement	8	For each variable of interest, give sources of data and details of methods of assessment (measurement). Describe comparability of assessment methods if there is more than one group		p. 10
Bias	9	Describe any efforts to address potential sources of bias		p. 10
Study size	10	Explain how the study size was arrived at		N/A
Quantitative variables	11	Explain how quantitative variables were handled in the analyses. If applicable, describe which		p. 10

		groupings were chosen, and why		
Statistical methods	12	(a) Describe all statistical methods, including those used to control for confounding<br>(b) Describe any methods used to examine subgroups and interactions<br>(c) Explain how missing data were addressed<br>(d) *Cohort study* - If applicable, explain how loss to follow-up was addressed<br>*Case-control study* - If applicable, explain how matching of cases and controls was addressed<br>*Cross-sectional study* - If applicable, describe analytical methods taking account of sampling strategy<br>(e) Describe any sensitivity analyses		p. 10
Data access and cleaning methods		..	RECORD 12.1: Authors should describe the extent to which the investigators had access to the database population used to create the study population.<br><br>RECORD 12.2: Authors should provide information on the data cleaning methods used in the study.	p. 10
Linkage		..	RECORD 12.3: State whether the study included person-level, institutional-level, or other data linkage across two or more databases. The methods of linkage and methods of linkage quality evaluation should be provided.	p. 10
**Results**				
Participants	13	(a) Report the numbers of individuals at each stage of the study (*e.g.*, numbers potentially eligible, examined for eligibility, confirmed	RECORD 13.1: Describe in detail the selection of the persons included in the study (*i.e.,* study population selection) including filtering based on data quality, data availability and linkage. The selection of	p. 2-3

		eligible, included in the study, completing follow-up, and analysed)<br>(b) Give reasons for non-participation at each stage.<br>(c) Consider use of a flow diagram	included persons can be described in the text and/or by means of the study flow diagram.	
Descriptive data	14	(a) Give characteristics of study participants (*e.g.*, demographic, clinical, social) and information on exposures and potential confounders<br>(b) Indicate the number of participants with missing data for each variable of interest<br>(c) *Cohort study* - summarise follow-up time (*e.g.*, average and total amount)		p. 2-3
Outcome data	15	*Cohort study* - Report numbers of outcome events or summary measures over time<br>*Case-control study* - Report numbers in each exposure category, or summary measures of exposure<br>*Cross-sectional study* - Report numbers of outcome events or summary measures		p. 2-3
Main results	16	(a) Give unadjusted estimates and, if applicable, confounder-adjusted estimates and their precision (e.g., 95% confidence interval). Make clear which confounders were adjusted for and why they were included<br>(b) Report category boundaries when		p. 2-3

		continuous variables were categorized<br>(c) If relevant, consider translating estimates of relative risk into absolute risk for a meaningful time period		
Other analyses	17	Report other analyses done—e.g., analyses of subgroups and interactions, and sensitivity analyses		p. 2-3
**Discussion**				
Key results	18	Summarise key results with reference to study objectives		p. 4-5
Limitations	19	Discuss limitations of the study, taking into account sources of potential bias or imprecision. Discuss both direction and magnitude of any potential bias	RECORD 19.1: Discuss the implications of using data that were not created or collected to answer the specific research question(s). Include discussion of misclassification bias, unmeasured confounding, missing data, and changing eligibility over time, as they pertain to the study being reported.	p. 5&7
Interpretation	20	Give a cautious overall interpretation of results considering objectives, limitations, multiplicity of analyses, results from similar studies, and other relevant evidence		p. 4,5 & 7
Generalisability	21	Discuss the generalisability (external validity) of the study results		p. 7
**Other Information**				
Funding	22	Give the source of funding and the role of the funders for the present study and, if		p. 12-13

		applicable, for the original study on which the present article is based		
Accessibility of protocol, raw data, and programming code		..	RECORD 22.1: Authors should provide information on how to access any supplemental information such as the study protocol, raw data, or programming code.	p. 12

Supplementary Data Table 7: Co-morbidities included as the covariates in the modelling

QCOVID risk group	Code
Atrial fibrillation	Q_DIAG_AF
Asthma	Q_DIAG_ASTHMA
Blood cancer	Q_DIAG_BLOOD_CANCER
Heart failure	Q_DIAG_CCF
Cerebral palsy	Q_DIAG_CEREBALPALSY
Coronary heart disease	Q_DIAG_CHD
Cirrhosis	Q_DIAG_CIRRHOSIS
Congenital heart disease	Q_DIAG_CONGEN_HD
COPD	Q_DIAG_COPD
Dementia	Q_DIAG_DEMENTIA
Diabetes type 1	Q_DIAG_DIABETES_1
Diabetes type 2	Q_DIAG_DIABETES_2
Epilepsy	Q_DIAG_EPILEPSY
Fracture	Q_DIAG_FRACTURE
Neurological disorder	Q_DIAG_NEURO
Parkinson's	Q_DIAG_PARKINSONS
Pulmonary hypertension	Q_DIAG_PULM_HYPER
Pulmonary rare	Q_DIAG_PULM_RARE
Peripheral vascular disease	Q_DIAG_PVD
Rheumatoid arthritis or SLE	Q_DIAG_RA_SLE
Respiratory cancer	Q_DIAG_RESP_CANCER
Severe mental illness	Q_DIAG_MENT_ILL
Sickle cell disease	Q_DIAG_SICKLE_CELL
Stroke/TIA	Q_DIAG_STROKE
Thrombosis or pulmonary embolus	Q_DIAG_VTE
Care housing category	Q_HOME_CAT
Learning disability or Down's	Q_LEARN_CAT
Kidney disease	Q_DIAG_CKD_LEVEL
More information on codes: https://github.com/EAVE-II/EAVE-II-data-dictionary Ref: Clift, A.K., et al. Living risk prediction algorithm (QCOVID) for risk of hospital admission and mortality from coronavirus 19 in adults: national derivation and validation cohort study. BMJ 371, m3731 (2020).	

Supplementary Data Table 8: Characteristics of people with severe obesity and normal BMI controls (SCORPIO study)

	Normal BMI controls (n=16)	PITCH normal BMI controls (V3D0 comparison) (n=25)	Severe obesity (n=28)	P value for normal BMI controls vs Severe obesity	P value for PITCH normal BMI controls and Severe obesity
**Age (mean, range in years)**	46 (38-60)	33 (21-59)	54 (37-69)	0.0870	<0.0001
**Sex (F/M)**	10/6	19/6	20/8	0.7376	0.7632
**BMI (mean, range in kg/m2)**	22.9 (18-25)	21.0 (18-25)	47.4 (38-67)	<0.0001	<0.0001
**Primary vaccination course (ChadOx1 vs BNT162b2)**	10/6	19/6	18/10	0.3791	0.3869
**Time after second dose of SARS-CoV-2 vaccination (mean, range in days)**	183 (140-239)	186 (155-223)	196 (182-215)	0.1531	0.2576
**Hypertension (number of individuals)**	0	1	15		
**Diabetes mellitus 2 (number of individuals)**	0	0	11		
		Diet controlled	3		
		Oral glucose lowering drugs	6		
		Insulin treatment	2		
**Cardiovascular disease (number of individuals)**	0	0	6		
**Non-alcoholic fatty liver disease (number of individuals)**	0	0	2		
**Obstructive sleep apnoea (number of individuals)**	0	0	8		

Groups were compared using ANOVA or non-parametric Kruskall-Wallis tests where appropriate.

Supplementary Data Table 9: Antibody dilutions in high-dimensional spectral flow cytometry

Surface mix					Total volume:	Overnight mix			Total volume:
**Peak Channel**	**Fluor**	**Marker**	**Dilution**	**ul to add**	**4400**	**Marker**	**Dilution**	**ul to add**	**4400**
**UV2**	BUV395					CD27	2000	2.2	
**UV6**	AF350					CD57	500	8.8	
**UV7**	BUV496					CD4	2000	2.2	
**UV8**	BUV563	FcRL5	500	8.800					
**UV9**	BUV615	CD19	2000	2.200	**FACS buffer:**				**perm buffer:**
**UV11**	BUV661				4400	CD11c	2000	2.2	4400
**UV14**	BUV737	CD10	1000	4.400					
**UV16**	BUV805					CD38	2000	2.2	
**V1/2**	eFluor450					Tbet	2000	2.2	
**V1/2**	BV421				**Samples**	ICOS	1000	4.4	**Samples**
**V4**	BV480	CD21	2000	2.200	40				40
**V6**	BV510					TCRgd	1000	4.4	
**V8**	SB570					CD45RA	500	8.8	
**V10**	BV605	Spike	500	8.800					
**V11**	BV650	CXCR3	500	8.800					
**V13**	BV711					GATA3	500	8.8	
**V14**	BV750					PD-1	1000	4.4	
**V15**	BV785	HLA-DR	2000	2.200	**Total stains:**				**Total stains:**
**B2**	KIRAVIA Blue 520				40	CD25	1000	4.4	40
**B3**	Alexa 532					IgM	1000	4.4	
**B3**	SparkBlue 550					CD3	2000	2.2	
**B8**	PerCP	CD14	2000	2.200					
**B9**	PerCPeFluor 710	CCR6	500	8.800					
**B9**	BB700	CD71	1000	4.400					
**B10**	BB790					IRF4	500	8.8	
**YG1**	PE	RBD	500	8.800					
**YG2**	SparkYG593					CD11b	2000	2.2	
**YG3**	AlexaFluor 594					CD44	1000	4.4	
**YG3**	PE-dazzle594	CXCR5	2000	2.200					
**YG4**	PE-AlexaFluor610	CD24	2000	2.200					
**YG5**	PE-Cy5	CXCR4	2000	2.200					
**YG7**	PE-Cy5.5					Foxp3	500	8.8	
**YG9**	PE-Cy7					RORGT	1000	4.4	
**YG10**	PE-Fire810	CCR7	500	8.800					
**R1**	APC	Spike	500	8.800					
**R2**	Alexa 647	RBD	500	8.800					
**R2**	Spark NIR685	CD20	2000	2.200					

**R4**	Alexa Fluor 700					Ki67	1000	4.4	
**R5**	ViaKrome808	Viability	2000	2.200					
**R7**	APC-Fire750	IgD	2000	2.200					
**R8**	APCFire810					CD8	2000	2.2	
	Blocking	Fc block	100	44.000		Fc block	100	44	

Supplementary Data Table 10: PITCH consortium

First Name	Middle Name(s)/Initial(s)	Last Name	Department (if appropriate)	Institution
**Eleanor**		Barnes	Nuffield Department of Medicine	University of Oxford
**Sagida**		Bibi	Oxford Vaccine Group, Department of Paediatrics	University of Oxford
**Miles**		Carroll	Nuffield Department of Medicine	University of Oxford
**Christopher**	P.	Conlon	Nuffield Department of Medicine	University of Oxford
**Thushan**	I.	de Silva	Department of Infection, Immunity and Cardiovascular Disease	University of Sheffield
**Alexandra**	S	Deeks	Nuffield Department of Medicine	University of Oxford
**Susan**	L	Dobson	Institute of Infection, Veterinary and Ecological Sciences	University of Liverpool
**Christina**		Dold	Oxford Vaccine Group, Department of Paediatrics	University of Oxford
**Susanna**		Dunachie	Nuffield Department of Medicine	University of Oxford
**Christopher**	JA	Duncan	Translational and Clinical Research Institute	Newcastle University
**Sian**		Faustini	Institute for Immunology and Immunotherapy, College of Medical and Dental Science	University of Birmingham
**Sarah**		Foulkes		UK Health Security Agency
**John**		Frater	Nuffield Department of Medicine	University of Oxford
**Victoria**		Hall		UK Health Security Agency
**Susan**		Hopkins		UK Health Security Agency
**Jasmin**		Islam		UK Health Security Agency
**Katie**		Jeffery	Radcliffe Department of Medicine	University of Oxford
**Paul**		Klenerman	Nuffield Department of Medicine	University of Oxford
**Barbara**		Kronsteiner	Nuffield Department of Medicine	University of Oxford
**Teresa**		Lambe	Oxford Vaccine Group, Department of Paediatrics	University of Oxford
**Stephanie**		Longet	Nuffield Department of Medicine	University of Oxford
**Alexander**	J.	Mentzer	Nuffield Department of Medicine	University of Oxford
**Shona**	C	Moore	Institute of Infection, Veterinary and Ecological Sciences	University of Liverpool
**Ashley**		Otter		UK Health Security Agency
**Rebecca**	P.	Payne	Translational and Clinical Research Institute	Newcastle University
**Alex Richter**		Richter	Institute for Immunology and Immunotherapy, College of Medical and Dental Science	University of Birmingham
**Sarah**	L.	Rowland-Jones	Department of Infection, Immunity and Cardiovascular Disease	University of Sheffield
**Donal**		Skelly	Nuffield Department of Clinical Neurosciences	University of Oxford
**Lizzie**		Stafford	Nuffield Department of Medicine	University of Oxford
**James**	E.D.	Thaventhiran	MRC Toxicology Unit	University of Cambridge
**Lance**		Turtle	Institute of Infection, Veterinary and Ecological Sciences	University of Liverpool
**Daniel**	G.	Wootton	Institute of Infection, Veterinary and Ecological Sciences	University of Liverpool
