# Supplementary material for: Accelerated waning of the humoral response to COVID-19 vaccines in obesity
Source: Nat Med. 2023 May 11;29(5):1146–54. doi: 10.1038/s41591-023-02343-2 (PMC10202802; doi:10.1038/s41591-023-02343-2)
Supplement: Supplementary file 2 — Reporting Summary [file 41591_2023_2343_MOESM2_ESM.pdf]

nature portfolio

Corresponding author(s): Sadaf Farooqi and James Thaventhiran

Last updated by author(s): 31/3/2023

Reporting Summary

Nature Portfolio wishes to improve the reproducibility of the work that we publish. This form provides structure for consistency and transparency in reporting. For further information on Nature Portfolio policies, see our Editorial Policies and the Editorial Policy Checklist.

Statistics

For all statistical analyses, confirm that the following items are present in the figure legend, table legend, main text, or Methods section.

n/a	Confirmed	
☐	☒	The exact sample size (*n*) for each experimental group/condition, given as a discrete number and unit of measurement
☐	☒	A statement on whether measurements were taken from distinct samples or whether the same sample was measured repeatedly
☐	☒	The statistical test(s) used AND whether they are one- or two-sided<br>*Only common tests should be described solely by name; describe more complex techniques in the Methods section.*
☐	☒	A description of all covariates tested
☐	☒	A description of any assumptions or corrections, such as tests of normality and adjustment for multiple comparisons
☐	☒	A full description of the statistical parameters including central tendency (e.g. means) or other basic estimates (e.g. regression coefficient) AND variation (e.g. standard deviation) or associated estimates of uncertainty (e.g. confidence intervals)
☐	☒	For null hypothesis testing, the test statistic (e.g. *F*, *t*, *r*) with confidence intervals, effect sizes, degrees of freedom and *P* value noted<br>*Give P values as exact values whenever suitable.*
☒	☐	For Bayesian analysis, information on the choice of priors and Markov chain Monte Carlo settings
☒	☐	For hierarchical and complex designs, identification of the appropriate level for tests and full reporting of outcomes
☒	☐	Estimates of effect sizes (e.g. Cohen's *d*, Pearson's *r*), indicating how they were calculated

Our web collection on statistics for biologists contains articles on many of the points above.

Software and code

Policy information about availability of computer code

Data collection	No software was used for data collection
Data analysis	R (version 3.6.1) was used to carry out all statistical analyses in EAVE II.<br><br>All code used in the EAVE II study is publicly available at https://github.com/EAVE-II/covid-obesity-humoral-response.<br><br>For the SCORPIO study, analysis was completed using GraphPad Prism software version 9.3.1.<br><br>tSNE, FlowSOM and heatmap analysis were performed using R (version 4.1.2) using code that has previously been described (Pasciuto, E. et al. Microglia Require CD4 T Cells to Complete the Fetal-to-Adult Transition. Cell 182, 625-640 e624, doi:10.1016/j.cell.2020.06.026 (2020).)<br><br>Manual gating of flow cytometry data was performed using FlowJo v10.8 software (Tree Star).

For manuscripts utilizing custom algorithms or software that are central to the research but not yet described in published literature, software must be made available to editors and reviewers. We strongly encourage code deposition in a community repository (e.g. GitHub). See the Nature Portfolio guidelines for submitting code & software for further information.

Data

Policy information about availability of data

All manuscripts must include a data availability statement. This statement should provide the following information, where applicable:

- Accession codes, unique identifiers, or web links for publicly available datasets
- A description of any restrictions on data availability
- For clinical datasets or third party data, please ensure that the statement adheres to our policy

The study uses established data security principles and processes to keep information secure.

The epidemiology study data that support the findings of this study are not publicly available because they are based on de-identified national clinical records. These are, however, available by application via Scotland's National Safe Haven from Public Health Scotland. The data used in this study can be accessed by researchers through NHS Scotland's Public Benefit and Privacy Panel via its Electronic Data Research and Innovation Service. EAVE II researchers do not have access to view personal medical records, and do not know the identities of any individuals. The information is grouped into broad categories and any information that could identify individuals is removed.

Anonymised data from the SCORPIO study have been included in the manuscript; stored samples are available from the corresponding authors on request.

Human research participants

Policy information about studies involving human research participants and Sex and Gender in Research.

Reporting on sex and gender

We have reported sex on the participants for both the EAVE II and SCORPIO study.

Population characteristics

The EAVE II cohort contains key information relevant to COVID-19 for almost 5.4 million individuals registered with a general practice (GP) in Scotland – approximately 98-99% of the Scottish population. The EAVE II surveillance platform includes information on clinical and demographic characteristics of each individual, their vaccination status and type of vaccine used and information on positive SARS-COV-2 infection and subsequent hospitalization or death.

Full population characteristics (age, sex, BMI, comorbidities) are described in Table 2 (EAVE II study) and Extended data Table 4 (SCORPIO study).

Recruitment

n/a for the EAVE II Cohort

For the SCORPIO study, participants were recruited from the obesity clinic in Addenbrooke's hospital, Cambridge. All patients in the obesity clinic were approached over the recruitment period of SCORPIO. As referral to the clinic is based on severity of obesity (BMI > 35 kg/m2 with one or more obesity-related comorbidities or BMI>40 kg/m2), this means that results obtained in this group may not be generalisable to individuals with mild or moderate obesity. People with acquired (HIV, immunosuppressant drugs) or congenital immune deficiencies and cancer were excluded which means results will not be generalisable to people with obesity and those conditions. Given the set up of multiple visits over a short period of time, participants with severe mobility issues due to their obesity, living at greater distance from the hospital, inflexible work or care commitments were less likely to consent to participate. The participants with normal BMI were recruited through advertisement in Cambridge University departmental emails and through the PITCH study which is a cohort of health care workers. Inclusion criteria were: no comorbidities, normal BMI and thus selected a relatively fit and healthy group.

Ethics oversight

1. EAVE II has been given approval from:

Ethical approval was granted by the National Research Ethics Service Committee, Southeast Scotland 02 (reference number: 12/SS/0201) for the study using the Early Pandemic Evaluation and Enhanced Surveillance of COVID-19 (EAVE II) platform. Approval for data linkage was granted by the Public Benefit and Privacy Panel for Health and Social Care (reference number: 1920-0279). Individual written patient consent was not required for this analysis.

2. The SCORPIO study has been given approval from:

1) Clinical studies in people with severe obesity and normal BMI controls were approved by the National Research Ethics Committee and Health Research Authority (East of England – Cambridge Research Ethics Committee (SCORPIO study, SARS-CoV-2 vaccination response in obesity amendment of ''NIHR BioResource'' 17/EE/0025)). Each participant provided written informed consent. All studies were conducted in accordance with the Declaration of Helsinki.

2) Additional normal BMI controls were recruited in Oxford, UK as part of the PITCH study under the GI Biobank Study 16/YH/0247, approved by Yorkshire & Humber Sheffield Research Ethics Committee, which was amended for this purpose on 8 June 2020.

3) For external validation of assays for neutralizing antibodies, a panel of serum samples from the C-VELVET study (approved by the West Midlands Solihull Research Ethics Committee, REC reference: 21/WM/0082, IRAS project ID: 296926) was tested.

Note that full information on the approval of the study protocol must also be provided in the manuscript.

Field-specific reporting

Please select the one below that is the best fit for your research. If you are not sure, read the appropriate sections before making your selection.

☒ Life sciences ☐ Behavioural & social sciences ☐ Ecological, evolutionary & environmental sciences

For a reference copy of the document with all sections, see nature.com/documents/nr-reporting-summary-flat.pdf

Life sciences study design

All studies must disclose on these points even when the disclosure is negative.

Sample size	Not applicable for EAVE II as this is a large population level based study;<br>N/A for SCORPIO as this is an exploratory case-control study.
Data exclusions	Data from participants in the SCORPIO study who tested positive for SARS-CoV-2 infection by RT-PCR was excluded. Missing data in addition to this were due to 1) occasional difficult venepuncture in people with obesity, 2) insufficient PBMCs were isolated for both T and B cell analysis or 3) insufficient sample to run both WT and omicron neutralization assays. Therefore, we have specified how many participants were included per analysis/ per figure.
Replication	This is not applicable for both studies. There is no similar cohort available to replicate the EAVE II studies. Similarly it was not possible to replicate the case-control SCORPIO study. Some of the immunogenicity markers were performed in technical duplicates (quantification of neutralising antibodies to SARS-CoV-2).
Randomization	The SCORPIO case-control study was not randomized. Severely obese cases and normal BMI controls were defined by BMI criteria.
Blinding	Researchers conducting immunoassays used samples that were fully anonymised and as such were blinded to the case vs control status of participants.

Reporting for specific materials, systems and methods

We require information from authors about some types of materials, experimental systems and methods used in many studies. Here, indicate whether each material, system or method listed is relevant to your study. If you are not sure if a list item applies to your research, read the appropriate section before selecting a response.

Materials & experimental systems

n/a	Involved in the study	
☐	☒	Antibodies
☐	☒	Eukaryotic cell lines
☒	☐	Palaeontology and archaeology
☒	☐	Animals and other organisms
☐	☒	Clinical data
☒	☐	Dual use research of concern

Methods

n/a	Involved in the study	
☒	☐	ChIP-seq
☐	☒	Flow cytometry
☒	☐	MRI-based neuroimaging

Antibodies

Antibodies used

The following antibodies were used:

BUV395 Mouse Anti-Human CD27, Clone L128, BD Biosciences, cat# 563816, AB_2744349
CD57 Antibody (TB01) [Alexa Fluor® 350], Clone TB01, Novus Biologicals, cat# NBP2-62203AF350, AB_2909528
Hu CD4 BUV496 M-T477 50ug, BD Biosciences, cat# 750175, AB_2874380
BUV563 Mouse Anti-Human FCRL5 (CD307e), Clone 509F6, BD Biosciences, cat# 749598, AB_2873900
BUV615 Mouse Anti-Human CD19, Clone HIB19, BD Biosciences, cat# 751273, AB_2875287
BUV661 Mouse Anti-Human CD11c, Clone B-ly6, BD Biosciences, cat# 612967, AB_2870241
BUV737 Mouse Anti-Human CD10, Clone HI10a, BD Biosciences, cat# 741825, AB_2871160
BUV805 Mouse Anti-Human CD38, Clone HB7, BD Biosciences, cat# 742074, AB_2871359
Brilliant Violet 421™ anti-human/mouse/rat CD278 (ICOS) Antibody, Clone C398.4a, Biolegend, cat# 313524, AB_2562545
T-bet Monoclonal Antibody (eBio4B10 (4B10)), eFluor™ 450, eBioscience™, Clone 4B10, Thermo Fisher Scientific, cat# 48-5825-82, AB_2784727
BV480 Mouse Anti-Human CD21, Clone B-ly4, BD Biosciences, cat# 746613, AB_2743893
BV510 Mouse Anti-Human TCR γδ, Clone B1, BD Biosciences, cat# 740179, AB_2739932
MOUSE ANTI HUMAN CD45RA:StarBright Violet 570, Clone F8-11-13, Bio-Rad, cat# MCA88SBV570, AB_871980
BV650 Mouse Anti-Human CD183, Clone 1C6/CXCR3, BD Biosciences, cat# 740603, AB_2740303
BV711 Mouse Anti-GATA3, Clone L50-823, BD Biosciences, cat# 565449, AB_2739242

BV750 Mouse Anti-Human CD279 (PD-1), Clone EH12.1, BD Biosciences, cat# 747446, AB_2872125
BV786 Mouse Anti-Human HLA-DR, Clone G46-6, BD Biosciences, cat# 564041, AB_2738559
BB515 Rat Anti-Human CXCR5 (CD185), BD Biosciences, cat#564624, AB_2738871
IgM Antibody (IM373) [Alexa Fluor® 532], Clone IM373, cat# NBP2-34650AF532, AB_2909529
Spark Blue™ 550 anti-human CD3 Antibody, Clone SK7, BioLegend, cat# 344851, AB_2819984
CD14 Monoclonal Antibody (61D3), PerCP-Cyanine5.5, eBioscience™, Clone 61D3, Thermo Fisher Scientific, cat# 45-0149-42, AB_1518736
CD196 (CCR6) Monoclonal Antibody (R6H1), PerCP-eFluor™ 710, eBioscience™, Clone R6H1, Thermo Fisher Scientific, cat# 46-1969-42, AB_10597900
BB700 Mouse Anti-Human CD71, Clone M-A712, BD Biosciences, cat# 746082, AB_2743458
IRF4-BB790, Clone Q9-343, BD Biosciences, custom conjugation
Spark YG™ 593 anti-mouse/human CD11b Antibody, Clone M1/70, BioLegend, cat# 101282, AB_2892261
Alexa Fluor® 594 anti-human CD44 Antibody, Clone C44Mab-5, BioLegend, cat# 397509, AB_2860987
PE/Dazzle™ 594 anti-human CD25 Antibody, Biolegend, Cat# 302646, AB_2734260
CD24 Monoclonal Antibody (SN3), PE-Alexa Fluor™ 610, Clone SN3, Thermo Fisher Scientific, cat# MHCD2422, AB_1468089
PE/Cyanine5 anti-human CD184 (CXCR4) Antibody, Clone 12G5, BioLegend, cat# 306508, AB_314614
FOXP3 Monoclonal Antibody (FJK-16s), PE-Cyanine5.5, eBioscience™, Clone FJK-165, Thermo Fisher Scientific, cat# 35-5773-82, AB_11218094
ROR gamma (t) Monoclonal Antibody (B2D), PE-Cyanine7, eBioscience™, Clone B2D, Thermo Fisher Scientific, cat# 25-6981-82, AB_2784671
PE/Fire™ 810 anti-human CD197 (CCR7) Antibody, Clone G043H7, BioLegend, cat# 353269, AB_2894572
Spark NIR™ 685 anti-human CD20 Antibody, Clone 2H7, BioLegend, cat# 302366, AB_2860775
Ki-67 Monoclonal Antibody (SolA15), Alexa Fluor™ 700, eBioscience™, Clone SolA15, Thermo Fisher Scientific, cat# 56-5698-82, AB_2637480
ViaKrome 808 Fixable Viability Dye, Beckman Coulter, cat# C36628
APC/Fire™ 750 anti-human IgD Antibody, Clone IA6-2, BioLegend, cat# 348238, AB_2616988
APC/Fire™ 810 anti-human CD8 Antibody, Clone SK1, BioLegend, cat# 344764, AB_2860890
Biotinylated detection mAb 7-B6-1, Mabtech, cat# 3420-4APT-10, Batch 56.3
Anti-human IgG Fc Specific – PE, Clone HP6043, Leinco Technologies, Inc., cat# I-127

Specific dilutions are included in supplementary table 9.

Validation

All antibodies used are commercially available antibodies and validated by the supplier.

Eukaryotic cell lines

Policy information about cell lines and Sex and Gender in Research

Cell line source(s)

Luminescent HEK293T reporter cells for SARS-CoV-2 were generated in the Matheson lab as previously described (https://doi.org/10.1371/journal.ppat.1010265). They are available from the National Institute for Biological Standards and Control (NIBSC, www.nibsc.org, catalogue number 101062).

Authentication

HEK293T cells were authenticated by STR profiling as previously described (https://doi.org/10.1371/journal.ppat.1010265).

Mycoplasma contamination

Luminescent HEK293T reporter cells for SARS-CoV-2 were regularly screened and confirmed to be mycoplasma negative (Lonza MycoAlert and IDEXX BioAnalytics).

Commonly misidentified lines
(See ICLAC register)

No commonly misidentified lines were used.

Clinical data

Policy information about clinical studies
All manuscripts should comply with the ICMJE guidelines for publication of clinical research and a completed CONSORT checklist must be included with all submissions.

Clinical trial registration

This is not a clinical trial and as such does not require trial registration. Below are the relevant ethical approvals for the different arms of the study.

Study protocol

This is not a clinical trial and has no trial protocol.

Data collection

The EAVE II surveillance platform drew on near real-time nationwide health care data for 5.4 million individuals (~99%) in Scotland. It includes information on clinical and demographic characteristics of each individual, their vaccination status and type of vaccine used and information on positive SARS-CoV-2 infection and subsequent hospitalization or death.

The SCORPIO study recruited people with severe obesity (class II/ III WHO criteria of BMI ≥40 kg/m2 or BMI ≥ 35 kg/m2 with obesity-associated medical conditions such as type 2 diabetes, hypertension) who attended the obesity clinic at Cambridge University Hospitals NHS Trust and had received two doses of SARS-CoV-2 vaccination (first and second dose of ChAdOX1 nCoV-19 or BNT162b2 610 mRNA) between December 2021 and May 2022. Clinical and immunological measurements were taken before the third dose booster vaccination, 8 (-3) days, 28 (+-7) days and 105 (+-7) days after vaccination.

Outcomes

EAVE II study outcomes: The cohort analysed for this study consisted of individuals aged 18 and over who were administered with at least two doses of BNT162b2 mRNA, ChAdOx1 nCoV-19 or mRNA-1273 vaccines. Follow-up began 14 days after receiving the second dose until Covid-19 related hospitalization, Covid-19 related death or the end of study period. The primary outcome of interest was severe Covid-19, which was defined as Covid-19 related hospital admission or death, 14 days or more after receiving the second

vaccine or booster dose.

Outcomes SCORPIO study: The measurement of humoral immunity using serology, live virus neutralisation capacity, B and T cell immunity were the primary outcomes in the SCORPIO study.

Flow Cytometry

Plots

Confirm that:

☒ The axis labels state the marker and fluorochrome used (e.g. CD4-FITC).

☒ The axis scales are clearly visible. Include numbers along axes only for bottom left plot of group (a 'group' is an analysis of identical markers).

☒ All plots are contour plots with outliers or pseudocolor plots.

☒ A numerical value for number of cells or percentage (with statistics) is provided.

Methodology

Sample preparation	Peripheral blood samples were acquired in lithium heparin tubes from participants in the SCORPIO study. Peripheral blood mononuclear cells (PBMCs) were isolated by layering over lymphoprep density gradient medium (Stemcell Technologies) followed by density gradient centrifugation at 800 x g for 20 minutes at RT. PBMCs were isolated and washed twice using wash buffer (1X PBS, 1% foetal calf serum, 2mM EDTA) at 400 x g for 10 minutes at 4°C. Isolated PBMCs were resuspended in freezing media, aliquoted and stored at -80°C for up to a week before being transferred to liquid nitrogen until use. For using in flow cytometry 1mL PBMC samples were defrosted in a 37°C water bath, and then immediately diluted into 9mL of pre-warmed RPMI+10% Fetal Bovine serum (FBS). Cells were washed twice with 10 mL of FACS buffer (PBS containing 2% FBS and 1mM EDTA). Cells were then resuspended in 500uL of FACS buffer and cell numbers and viability were determined using a Countess™ automated cell counter (Invitrogen). 5×106 viable cells were transferred to 96-well plates for antibody staining. Cells were then washed once with FACS buffer, and stained with 100 µL of surface antibody mix (including B cell probes) for 2 hours at 4°C. Cells were then washed twice with FACS buffer, and fixed with the eBioscience Foxp3/Transcription Factor Staining Buffer (ThermoFisher #00-5323-00) for 30 min at 4°C. Cells were then washed with 1x eBioscience Foxp3/ Transcription Factor Permeabilisation buffer (ThermoFisher #00-801 8333-56) twice and stained with intracellular antibody mix in permeabilisation buffer at 4°C overnight. Following overnight staining, samples were washed twice with 1x permeabilisation buffer and once with FACS buffer before analysis.
Instrument	CytekTM Aurora
Software	FlowJo v10.8 software
Cell population abundance	Not applicable as no cell sorting was used.
Gating strategy	Data presented as % of total lymphocytes or % of B cells.<br>Prior gating strategy: Live (viability dye negative), singlets (fsc-a / fsc-h), B cells (CD19+ CD20+)<br>Extended data figures 3 and 6 contain pre-gating + full gating strategies for the above populations, as well as circulating TFH full gating strategy.

☒ Tick this box to confirm that a figure exemplifying the gating strategy is provided in the Supplementary Information.
